# Integrative *in silico* and *in vitro* screening of small molecules targeting RNA

**DOI:** 10.1002/cpz1.70431

**Published:** 2026-08-20

**Authors:** Sabrina Toews, Megan Ken, Harald Schwalbe

**Affiliations:** ^1^ Institute for Organic Chemistry and Chemical Biology Goethe University Frankfurt Frankfurt am Main, Hesse Germany; ^2^ Center of Biomolecular Magnetic Resonance (BMRZ) Goethe University Frankfurt Frankfurt am Main, Hesse Germany; ^3^ Department of Integrative Structural and Computational Biology The Scripps Research Institute La Jolla California

**Keywords:** docking, drug targeting, RNA, screening, small molecules

## Abstract

Targeting structured RNA elements with small molecules has emerged as a promising yet technically challenging strategy for antiviral drug discovery. Here, we present a comprehensive and experimentally validated workflow for the integrative *in silico* and *in vitro* screening of RNA‐binding small molecules. The approach is exemplified using conserved RNA elements from the SARS‐CoV‐2 genome, including the 5’‐terminal stem‐loop 1 and the programmed –1 ribosomal frameshift pseudoknot, but is broadly applicable to other structured RNAs. The workflow integrates high‐resolution RNA structural ensemble generation with virtual screening (VS) and nuclear magnetic resonance (NMR)‐based experimental validation. Conformational ensembles generated by fragment‐assembly approaches serve as targets for docking chemically diverse fragments and lead‐like libraries. Top‐ranked compounds are prioritized through consensus scoring and evaluated using ligand‐ and RNA‐observed NMR experiments to confirm binding, characterize interaction modes, and assess specificity. Such ranking allows for NMR‐guided fragment optimization, which enables systematic improvement of solubility, affinity, and selectivity through iterative medicinal chemistry in the pharmaceutical pipeline. Detailed procedures are provided for library preparation, ensemble‐based VS, hit validation, data interpretation, and progression toward functional assays, together with practical considerations, quality‐control parameters, and troubleshooting guidance to ensure reproducibility. By combining computational and experimental strategies that select for high‐specificity ligands within a unified framework, this set of protocols accelerates the discovery and optimization of RNA‐targeting small molecules and provides a scalable platform for RNA‐focused drug discovery. © 2026 The Author(s). *Current Protocols* published by Wiley Periodicals LLC.

**Basic Protocol 1**: Target and library preparation for virtual screening

**Basic Protocol 2**: Ensemble‐based virtual screening of low‐molecular‐weight compounds against RNA targets

**Basic Protocol 3**: Comparative hit prioritization and selectivity filtering for RNA‐binding small molecules

**Basic Protocol 4**: Preparation of RNA samples for *in vitro* validation of small‐molecule binding

**Basic Protocol 5**: NMR‐based *in vitro* screening of RNA–small molecule interactions

**Support Protocol**: Preparation of ligand stocks and NMR‐based quality control

**Basic Protocol 6**: Characterization and prioritization of validated RNA‐binding hits

## INTRODUCTION

### Structured RNA Elements as Central Regulators of Cellular Function

RNA molecules play a crucial role in cellular recognition, extending far beyond their traditional function of mediating communication between DNA and proteins (Ille et al., 2022). Transcriptomic studies have revealed that most of the human genome is transcribed but that only the minority of the resulting RNA is actually further translated into functional proteins. These findings highlight the vast regulatory landscape governed by coding and non‐coding RNAs. *Cis*‐acting RNAs are embedded within transcripts and are often involved in various biological processes, which include transcription and/or translational regulation, RNA processing, and catalysis of chemical reactions (Tants & Schlundt, 2024). These RNA elements are often found in untranslated regions (UTRs) or coding sequences of messenger RNA (mRNA) as well as in viral genomic and subgenomic RNA. Their regulatory functions originate from the presence of diverse RNA structural motifs. Regulatory RNA elements can feature several characteristic structural motifs, including stem‐loops (SLs), internal bulges, and more complex folds, like G‐quadruplexes, riboswitches, and pseudoknots (PKs) (Ceylan et al., 2025; Liu et al., 2022; Rückriegel et al., 2025; Toews et al., 2024). The structural features provide binding interfaces for proteins, endogenous or exogenic small molecules, and other RNA molecules, which function as *trans*‐acting factors in this context. Consequently, RNA structures and their interactions are increasingly recognized as central nodes in cellular regulatory networks and as potential therapeutic targets.

### RNA Elements as Targets in Drug Discovery

Despite their biological importance, RNAs have long been considered a challenging target for small‐molecule drug discovery (Kang et al., 2026). Additionally, most of the common techniques applied within drug discovery campaigns are well established for proteins but not for RNA. However, RNA targeting has attracted increasing attention as advances in structural and chemical biology have expanded our understanding of RNA architecture and function (Shao & Zhang, 2020; Zafferani & Hargrove, 2021). RNAs are an intriguing class of therapeutic targets due to their intrinsic structural dynamics and relatively limited chemical diversity compared to proteins, which could enable highly specific ligand recognition in principle. However, these same properties complicate drug discovery efforts. Unlike proteins, which typically have well‐defined binding pockets, RNA molecules often adopt flexible conformational ensembles and have a negatively charged phosphate backbone that limits the number of well‐defined, selective binding sites for small molecules (Ganser et al., 2019). Additionally, many functional RNA elements dynamically interconvert between alternative structural states, which further complicates the computational prediction of ligand‐binding sites and the experimental identification of selective binders (Stelzer et al., 2011). Consequently, despite their functional importance in numerous biological processes, structured RNAs remain comparatively understudied as small‐molecule drug targets. Although the discovery of small molecules binding to structured RNA elements is a critical first step, RNA binding alone is not sufficient to demonstrate functional modulation or therapeutical potential. Hit compounds must recognize the target RNA motif with appropriate affinity and selectivity, engage the target in its native environment, induce changes in RNA structure or interactions, and alter RNA‐dependent outputs in a way that matches a proposed mechanism of action (Disney, 2019; Falese et al., 2021; Childs‐Disney et al., 2022). Therefore, distinguishing RNA binding from actual RNA modulation is essential and requires an integrated view that connects biophysical data to structural remodeling, changes in RNA processing or translation, and downstream phenotypic or pharmacological effects.

### Problems with Methods Used in RNA‐Targeted Drug Discovery

Numerous experimental and computational approaches have been developed to interrogate RNA structure and RNA‐ligand interactions, including high‐throughput screening (HTS), structural biology, and structure‐guided design strategies (Cai et al., 2025; Costales et al., 2020; Meyer et al., 2020). However, many of these methods capture only a subset of the diverse behavior of RNAs. For example, structural techniques such as crystallography or cryo‐electron microscopy (cryo‐EM) provide detailed snapshots of low‐energy conformations but may not capture the full structural diversity of dynamic conformational ensembles. Moreover, their application to RNA systems presents additional challenges. RNAs are inherently flexible and frequently adopt multiple conformational states, making it difficult to obtain a single well‐defined structural representation (Bonilla & Jang, 2024). Many regulatory RNA elements are relatively small and lack extensive tertiary architecture, which complicates crystallization and can limit structural resolution in cryo‐EM studies. In addition, RNA structures are highly sensitive to environmental conditions such as ion concentrations, the presence of other biomacromolecules or ligands that can bind RNA, and temperature, which can shift conformational equilibria and influence experimental outcomes. Consequently, although these approaches provide valuable structural insights, they often fail to capture the dynamic and heterogeneous nature of functional RNA structures. Together, these limitations highlight the need for complementary strategies that integrate computational and experimental approaches to efficiently explore RNA structural landscapes and identify small molecules capable of targeting functional RNA elements.

### Conceptual Foundation of an Integrative Screening Strategy

To address these challenges, we developed an integrative strategy that combines ensemble‐based VS (EBVS) with targeted experimental validation to efficiently identify small molecules that bind structured RNA elements (Toews, Ceylan, et al., 2025). In this workflow, large chemical libraries are first screened *in silico* against structural ensembles of RNA targets, allowing rapid evaluation of tens of thousands of compounds while accounting for the intrinsic conformational flexibility of RNA. This use of RNA ensembles in VS has previously been shown to significantly increase performance in comparison to docking single structures (Ganser et al., 2018). The resulting docking results are then subjected to stringent computational filtering and comparative analysis, including selectivity assessment against additional decoy RNA structures, to eliminate likely nonspecific binders and drastically reduce the number of candidate molecules. This multistage *in silico* triaging step enables the prioritization of a small subset of high‐confidence compounds before any experimental work is performed. These selected candidates are subsequently evaluated using nuclear magnetic resonance (NMR) spectroscopy–based binding assays, enabling direct detection and characterization of RNA–small molecule interactions *in vitro*. A detailed protocol on how RNA‐targeted NMR screening can be performed and evaluated was previously published by the group of Qi Zhang but does not include previous filtering of a set of different RNAs as input and numerous small molecules (Thompson et al., 2019). The approach presented here provides an efficient and scalable strategy for discovering ligands targeting structured RNA regulatory elements while minimizing the experimental burden associated with large‐scale screening campaigns.

### Computational and Experimental Data Outputs from RNA‐Targeted Ligand Screening

The integrative RNA‐targeted screening workflow produces diverse, cross‐validated data. Computational outputs include docking scores, predicted ligand poses, and selectivity filters across RNA conformations. Experimental data provide NMR‐based ligand and target engagement signatures, including chemical shift perturbations (CSPs) and line‐broadening patterns. These datasets together yield ranked hit lists, preliminary binding affinities, site‐specific interaction maps, and early structure‐activity relationships (SARs), highlighting that RNA binds and engages with ligands *in vitro*. However, they primarily capture the early stages of the discovery process: confirmation of binding, localization of binding sites, and characterization of basic SARs. Establishing that a given ligand truly modulates RNA function requires downstream assays. These assays may include reporter systems, splicing or translation readouts, RNA stability measurements, viral replication assays, phenotypic rescue experiments, and cellular target‐engagement or structure‐probing methods. A detailed treatment of these functional and cellular assays is beyond the scope of the protocols described here, and we instead refer readers to recent reviews that comprehensively discuss how RNA‐binding ligands are linked to changes in RNA function and mechanism of action (Childs‐Disney et al., 2022; Koehn et al., 2023; Veenbaas et al., 2025). Cross‐referencing computational predictions with experimental observations allows us to identify functional RNA motifs, characterize binding specificity, and prioritize candidate small molecules for further biochemical or cellular validation.

### Viral RNA Elements as Motivating Examples

Functional RNA elements within viral genomes constitute attractive targets for antiviral drug discovery, as small molecules binding to conserved RNA structural motifs can disrupt interactions and conformational dynamics required for viral replication (Hermann, 2016). We have therefore established our integrative screening technique using published Fragment Assembly of RNA with Full‑Atom Refinement 2 (FARFAR2) ensemble data for the viral severe acute respiratory syndrome coronavirus 2 (SARS‐CoV‐2) RNA genome (Rangan et al., 2021). Although the FARFAR2 dataset contains structural ensembles for numerous RNA elements across the viral genome, the application of pre‐filtering in our VS workflow enabled us to narrow the analysis to two *cis*‐acting RNA elements for detailed characterization of potential RNA‐ligand interactions (Toews, Ceylan, et al., 2025). These elements differ completely in their structural features. The SL1, located within the 5’‐UTR of the viral RNA genome, is a short hairpin RNA (here, 29 nucleotides) including an asymmetrical 1 × 2 loop. In contrast, the PK is larger (here, 69 nucleotides) and part of a complex frameshift machinery that enables alternative translation of the viral genome (Kelly et al., 2021; Richter et al., 2021; Wacker et al., 2020).

### Opportunities in and Limitations of Computationally Guided RNA‐Ligand Screening

**Figure 1:**
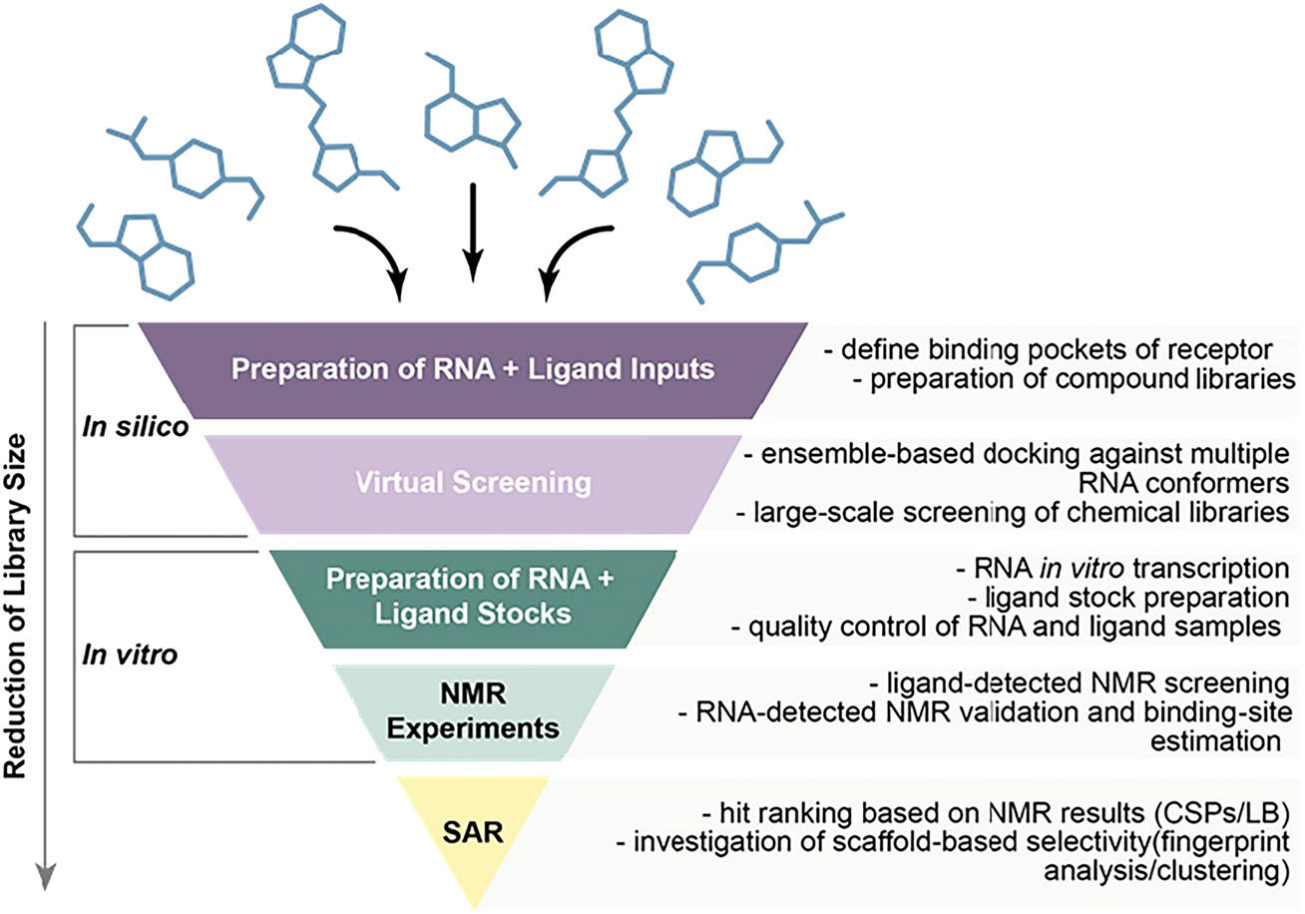
From volume to value: results‐focused library reduction. This is the overarching strategy of the integrated screening approach. The procedure is initiated with the preparation of the RNA and ligand inputs for *in silico* docking. Subsequently, the approach transitions to ensemble‐based virtual screening against a multitude of RNA conformers. Moving to experimental validation, the RNA and ligand stocks must be prepared. Following this, NMR experiments are performed for binding validation, resulting in the final step: structure‐activity relationship (SAR) analysis of the tested compounds.

Compared with traditional screening strategies, the integrative workflow presented here places strong emphasis on extensive computational filtering prior to experimental validation (Fig. 1). EBVS of large compound libraries against structured RNA elements enables rapid evaluation of tens of thousands of small molecules and allows early identification of candidates with favorable binding characteristics (Ganser et al., 2018). Subsequent ranking and selectivity filtering steps, including comparative screening against additional RNA structures, drastically reduce the number of potential hits and remove compounds likely to act as nonspecific RNA binders. This multistage computational triaging substantially narrows the candidate space before any *in vitro* experiments are performed, thereby focusing experimental efforts on a small set of high‐confidence molecules. As a result, downstream validation experiments such as NMR‐based binding assays can be performed more efficiently and with higher success rates. However, despite the power of computational prioritization, *in silico* predictions alone cannot fully capture the complexity of RNA structural dynamics and ligand interactions, making experimental validation essential for confirming true RNA‐binding compounds. Although ensemble‐based docking substantially improves upon single‐structure approaches by better accounting for RNA flexibility, it still operates under approximations in conformational sampling, force fields, and scoring and may miss or underweight low‐population, functionally relevant conformers (Korb et al., 2012; Zhou et al., 2022). In our workflow, we therefore explicitly use EBVS as a powerful prioritization and pocket‐hypothesis tool whose predictions are subsequently refined and validated by NMR‐based binding assays, rather than a defined measure of modulation.

To strengthen the link between computational pocket prediction and experimental validation further, covalent target‐engagement methods such as Chemical Cross‐Linking and Isolation by Pull‐down (Chem‐CLIP) and competitive Chem‐CLIP (C‐Chem‐CLIP), as well as related photoaffinity‐based strategies and in‐cell structural profiling approaches, provide powerful nucleotide‐resolved readouts of ligand‐binding sites on RNA (Bereiter et al., 2025; Costales et al., 2020; Disney, 2019; Shah et al., 2025). In Chem‐CLIP and C‐Chem‐CLIP, the ligands are equipped with crosslinking and enrichment handles. This enables the covalent capture of RNA‐ligand complexes in cells, followed by sequencing‐based mapping of the engaged nucleotides (Tong et al., 2025). This process has been extended to Chem‐CLIP fragment mapping (Chem‐CLIP‐Frag‐Map) for the systematic definition of binding pockets and the assembly of higher‐affinity ligands (Suresh et al., 2020). Complementary methods, such as Frag‐MaP, which combines fully functionalized fragment probes with mutational profiling, compare reactivity patterns between ligand‐free and ligand‐bound states in order to identify protected or allosterically perturbed nucleotides. This process validates or refines computationally predicted pockets directly within their native structural context. Integrating these covalent and chemical‐probing readouts with ensemble‐based docking results enables rigorous confirmation of predicted binding sites and delineation of precise RNA contact residues. It also allows for the iterative optimization of the SARs of RNA‐targeted ligands within a single, rational discovery workflow.

### Inclusive Protocols Needed for an Integrative RNA Screening Approach

**Figure 2:**
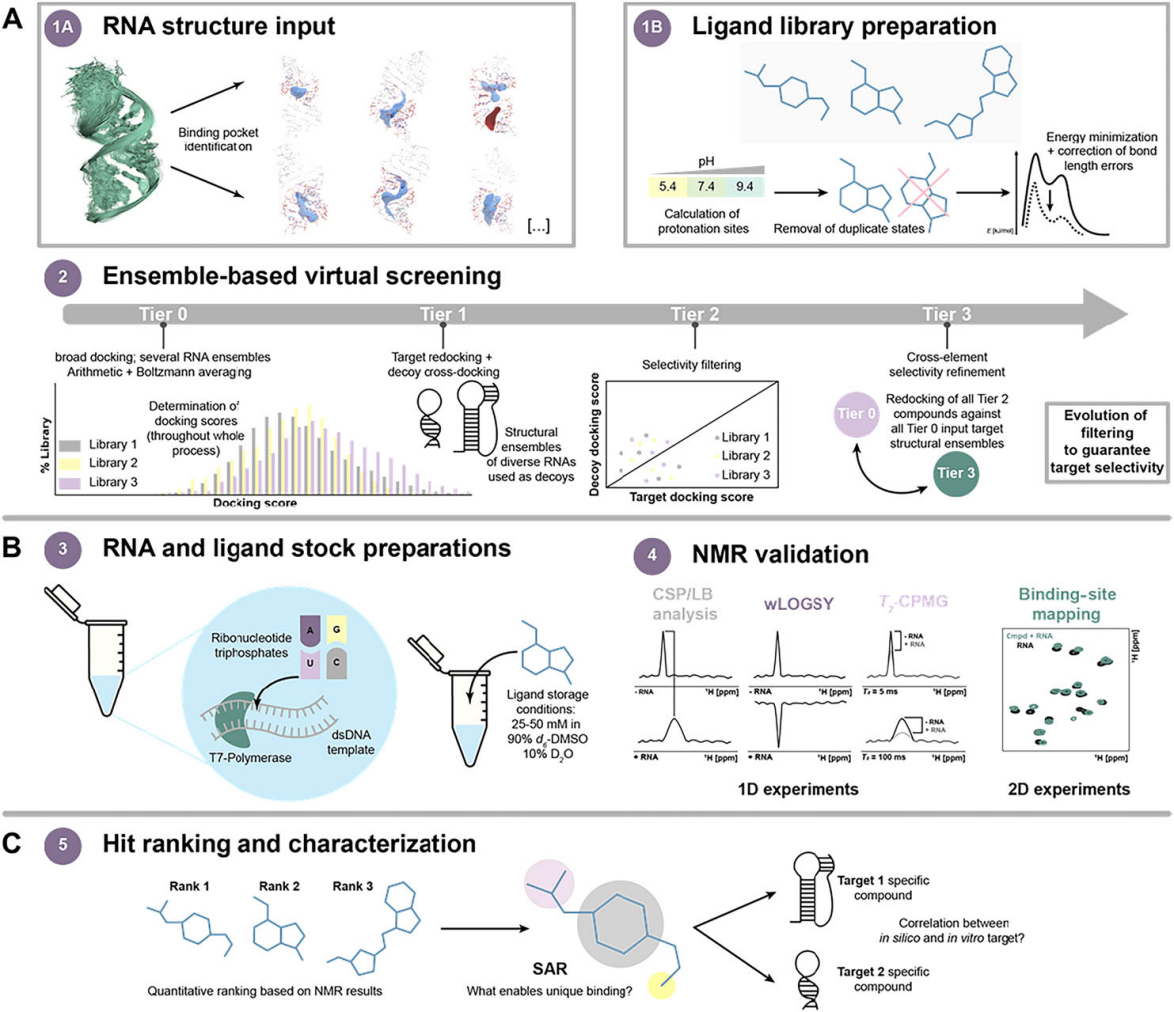
Overview of the integrative *in silico* and *in vitro* RNA‐targeted screening workflow. **(A)** Panel 1A shows the generation of RNA conformational ensembles for docking and the identification of possible binding pockets. Panel 1B depicts ligand preparation, including calculation of the protonation state across pH levels of 5.4, 7.4, and 9.4 and deduplication of chemically redundant species. Tiered virtual screening and selection are shown in 2. This progresses from the full library (Tier0) through successive filtering to enriched, high‐priority compounds (Tier3). **(B)** Prior to performing actual *in vitro* experiments, the target RNA(s) and ligand stocks need to be prepared (3). To ensure the integrity of these stocks, they should be routinely evaluated via NMR. Actual experiments for the identification of any possible interaction include a specific set of 1D proton‐detected NMR experiments (1D‐^1^H, waterLOGSY, *T_2_*‐CPMG), which can be followed by 2D NMR experiments allowing binding‐site estimation when detected on the target biomolecule (4). **(C)** NMR experiments are further analyzed, and results are quantified, allowing ranking of the hits. This is followed by SAR analysis, including scaffold‐based clustering and ligand prioritization based on observed binding properties.

This article provides detailed protocols for implementing an integrative workflow for the discovery and validation of small molecules targeting structured RNA elements (Fig. 2). The procedures guide researchers through key steps of the pipeline, including the selection and preparation of available RNA structural ensembles for computational screening, EBVS of small‐molecule libraries, and computational prioritization of candidate ligands through comparative analysis and selectivity filtering. Subsequent protocols describe the preparation of RNA samples for experimental validation and the use of NMR spectroscopy to detect and characterize RNA–small molecule interactions *in vitro*. Finally, guidance is provided for analyzing experimental results and prioritizing validated RNA‐binding compounds for further study. Together, these protocols establish a practical framework that integrates computational and experimental approaches to identify and characterize ligands targeting structured RNA regulatory elements. In brief, Basic Protocol 1 outlines the preparation of RNA targets and small molecules for EBVS, and Basic Protocol 2 describes the actual EBVS procedure itself and the initial filtering of candidate ligands. Basic Protocol 3 outlines follow‐up comparative hit prioritization and selectivity filtering. Basic Protocol 4 covers the preparation of RNA, which is then required for Basic Protocol 5. The latter describes the NMR‐based screening of RNA−small molecule interactions. This is followed by Basic Protocol 6, which presents the characterization and prioritization of the *in vitro*−validated RNA‐binding hits. Finally, the Support Protocol describes the preparation of ligand stocks and NMR‐based quality control (QC) prior to the actual NMR screening experiments.

## STRATEGIC PLANNING

The protocols presented here provide an overview of an integrated screening approach that includes *in silico* pre‐filtering and *in vitro* cross‐validation. Before implementing the approach experimentally, data describing the 3D structure of the desired target RNA must be available. Our approach relies on 3D coordinates in the form of FARFAR2 models. One must also consider the availability of computing capacity, software packages, and virtual ligand libraries. Used libraries should always consist of ligands that can be purchased after prioritization based on *in silico* screening results for follow‐up *in vitro* experiments.

*NOTE*: Experiments involving RNA require careful technique to prevent contamination.

## Basic Protocol 1 TARGET AND LIBRARY PREPARATION FOR VIRTUAL SCREENING

This protocol outlines the preparation of RNA targets and small‐molecule libraries for EBVS via molecular docking. The goal is to create high‐quality binding‐site models with defined, druggable pockets and chemically optimized ligands that are suitable for computational docking studies. Proper execution of this protocol ensures reproducibility, accurate pocket identification, and comprehensive coverage of ligand chemical space. EBVS requires structurally accurate 3D structures and chemically consistent ligands; we have used published FARFAR2 models in our initial approach. RNA molecules often adopt multiple conformations, and small molecules may exist in different protonation states depending on the local pH level. Considering these factors increases the likelihood of identifying true binding interactions and reduces false positives in docking results. Successful execution of Basic Protocol 1 should result in i) RNA target ensembles with well‐defined docking pockets for each conformer; ii) ligand libraries that are chemically optimized; and iii) fully prepared inputs for reliable VS, supporting accurate identification of potential RNA‐targeting small molecules.

If experimental structural data are available, the accuracy of FARFAR2‐derived RNA models can be validated using additional techniques, such as residual dipolar couplings (RDCs) and small‐angle X‐ray scattering (SAXS) (Shi et al., 2020). The agreement between back‐calculated RDCs and theoretical SAXS profiles from the generated models or ensembles and the experimental data can be assessed. RDCs provide information on local bond vector orientations, whereas SAXS provides information on the global shape and dimensions of the RNA. Consistent agreement between the two datasets supports the structural relevance of the selected models, whereas discrepancies may indicate the need for further refinement or increased conformational sampling.

### Materials

#### Hardware

Platforms (operating systems running on specific chip architecture); full list of supported platforms available at https://www.molsoft.com/products.html

##### Software

Internal Coordinate Mechanics (ICM) version 3.9, Molsoft (https://www.molsoft.com/index.html), as docking software (here used for target and ligand preparation)
ChemAxon software version 19.21.0 (https://www.certara.com/chemaxon/) for calculation of protonated ligand states for energy optimization and ICM formatting
OpenBabel software version 3.1.1 (https://openbabel.org/) for removal of ligand duplicate states
PyMOL open GL software version 2.1 (https://www.pymol.org/) for visualization of structures

##### Files

Ligand libraries for commercially available compounds for VS (50k Diversity and RNA‐focused libraries, Life Chemicals, https://lifechemicals.com/)
FDA‐approved library (ZINC15, https://zinc15.docking.org/)
FARFAR2 RNA ensemble (https://github.com/DasLab/FARFAR2‐SARS‐CoV‐2; Rangan et al., 2021)

#### Target preparation

1 Download available FARFAR2 RNA structural ensembles from the respective FARFAR2 repository or generate 3D ensemble models using any RNA structure prediction program; use experimentally determined 3D models if available.
  Here, we used the published FARFAR2 RNA ensembles for the SARS‐CoV‐2 RNA genome (Rangan et al., 2021). This target input contains seven structural ensembles of diverse RNAs present within the viral genome. Our primary target ensemble was “PK” (frame‐shifting pseudoknot), but we cross‐docked our libraries to all available ensembles, including 5SL1, 5SL2, 5SL4, 5SL5, 3SL1, and 3SL2.
2 Extract the top‐scoring conformers of each cluster in the target RNA ensemble.
  In the GitHub repository for the FARFAR2‐SARS‐CoV‐2 ensembles, many models that were subsequently clustered are present as individual .pdb files, with the lowest numbers representing the lowest‐energy states. To optimize diversity and accuracy, we chose to use the lowest‐scoring .pdb file from each cluster. For the PK element, this was c.1.1.pdb, c.2.1.pdb, c.3.1.pdb, c.4.1.pdb, c.5.1.pdb, c.6.1.pdb, c.7.1.pdb, c.8.1.pdb, c.9.1.pdb, and c.10.1.pdb.
3 Open each conformer in PyMOL to visually inspect the structure. Check that no atoms are missing in the RNA backbone, check proper nucleotide connectivity, and look for any steric clashes.
  The following steps in the rest of the “Target preparation” section are to be performed on individual structures, repeating for each conformer model within an ensemble, for all ensembles.
4 Launch the ICM software graphical user interface (GUI) on the computer and load the structure file (.pdb).
5 Convert the structure file into an ICM object:
  a. Select the structure.
  b. Click MolMechanics → ICM Convert → Protein.
  c. Check the following options: delete water, optimize hydrogens, replace original, and display result. Leave all other options unchecked.
  d. Click OK to save the RNA structure as an ICM object.
    The 3D structural model converted to an ICM object does not yet constitute the required file type for executing docking. To dock, a portion of the ICM object must be defined as the region in which docking will occur, or the “receptor.” In more general terms, this would be called a “pocket.” There are several ways to define receptors using ICM software. We chose to use their PocketFinder function, but pockets can also be defined as a specified distance from a ligand, a list of specific residues, or a user‐defined box. Using PocketFinder, the pocket's shape will be determined by an ICM function that accounts for physical properties that include but are not limited to volume, solvent‐accessible surface area, and number of hydrogen‐bond donors and acceptors. These properties are then used to calculate a “druggability score,” which indicates how likely a given pocket is to be able to favorably bind a ligand. The user can toggle the “tolerance” of the PocketFinder algorithm, which will change the size and number of pockets generated. In our study, we always used the default value of 4.6. The user cannot change the shape of the pocket but can define the cutoff distance from the pocket, within which all atoms will be included in the receptor.
6 Generate the receptor:
  a. Identify binding pockets:
    i. Run the ICM package PocketFinder via Tools → PocketFinder.
    ii. Use default parameter and a tolerance value of 4.6.
    iii. Use the following decision criteria: Single or multiple pocket(s) detected → proceed with both. No pocket detected → discard conformer.
      Multiple pockets yield multiple receptors for a given conformer to maximize docking opportunities.
  b. For each pocket detected within the conformer, proceed individually to create the receptor as follows:
    i. Docking → New Project.
    ii. Name the docking project (receptor) systematically in a way that defines this receptor both by the conformer number and by the pocket number.
      This will be the prefix of all files generated that define the receptor.
      The naming convention we used was RNAname_conformer#_pocket#. An example of this is PK_1_1, where “PK” is the name of the RNA (pseudoknot), the first “1” corresponds to the conformer number used to generate this receptor, and the second “1” corresponds to the first pocket identified in this conformer.
    iii. Choose Define Sites Around Mesh.
    iv. From the dropdown menu, pick the mesh from the PocketFinder result that the receptor is being created around.
    v. Uncheck Make Receptor Maps Immediately.
    vi. Choose OK.
    vii. Adjust the probe position if desired (probe = four purple spheres). To continue, choose Go.
      The probe represents the initial position where sampling will begin and should usually be in the center of the binding pocket. We always kept the default position.
    viii. Adjust the size of the purple box if desired. To continue, choose Go.
      The purple box represents the region in which maps will be generated. We always kept the default position.
7 Make receptor maps:
  a. Docking → (Re) Make Receptor Maps.
  b. In the box that pops up, check the option Atom Occupancy Weighted. Keep the Max VW repulsion at the default value of 4.0.
    This will generate the receptor files: 10 different files with the filename prefix defined in the previous step (see step 6b) when you named the docking project, in the folder you specified at that time. These files will all have the same prefix (receptor name), followed by the unique suffixes gb.map, gc.map, ge.map, gh.map, gl.map, gs.map, LOG.htm, probe.ob, rec.ob, and .dtb.
8 Set up docking preferences by clicking Docking → Preferences→ General. In the box that pops up, do the following:
  a. Keep Flexible ring sampling level at 2.
  b. Keep Relax covalent geometry checked.
  c. Change Charge groups to auto.
  d. Keep everything else unchecked.
    Once this is done, all the receptor files will be generated with the filename prefix defined in step 6b, when you named the docking project (i.e., RNAname_conformer#_pocket#), in the folder you specified at that time. All these files will need to be present for each receptor to proceed with screening.

#### Library preparation

9 Download ligand libraries of interest that include commercially available molecules as .sdf files.
  Here, we used diversity and RNA‐focused libraries from https://lifechemicals.com/ and FDA‐approved compounds from the ZINC15 database using the “fda‐only” filter.
10 Load the ChemAxon program in the command line and make sure you have the Protonation Plugin loaded.
11 To generate a version of each library at pH 7.4, run the following command:


cxcalc ‐o "Library‐name"_ph7_4.sdf majorms ‐H 7.4 "Library‐name".sd ‐f sdf ‐M true ‐K true

12 To generate versions of the library at different pH values, replace the instances of “7.4” and “7_4” in the command above with the new pH.
  We did this at pH 5.4, 7.4, and 9.4.
  This step will output new .sdf files of your library, with all ligands represented by their protonation state at the specified pH, given their pKa values as predicted by the ChemAxon software. Including protonation states at three different pH values allows the reflection of potential experimental conditions and/or microenvironments that would cause a ligand to interact with an RNA at non‐physiological pH.
13 Remove duplicate structures:
  a. Download OpenBabel and open the command line.
  b. To re‐label the molecules themselves within the .sdf files to have a pH tag so which pH is the remaining state will be clear later, run the following command:


babel infile.sdf outfile.sdf --addtotitle “xx”

    Here, your infile is a library file at a certain pH. This file contains within it the ligand names from the originally downloaded file. All the library files at different pH's have the same ligand name labels. You will need to concatenate all these files to remove redundant protonation states, and if you do not change the names of the molecules themselves, you will not know which pH state(s) are the one(s) remaining. This step creates an outfile where the ligand name labels in the library files themselves are changed to be appended with whatever text you choose to put in the “xx” position. We did “ph5”, “ph7”, and “ph9” for our libraries determined at pHs 5.4, 7.4, and 9.4 respectively.
  c. Repeat for all protonation states.
  d. Concatenate all protonation state library files that have the ligand name changes.
  e. To remove redundant structures, run the following command:


babel infile.sdf outfile.sdf --unique [param]

    Here, your infile is the concatenated library file with all pH states. Your outfile will be a library file where duplicates are removed; –unique = tells OpenBabel to skip any molecule that is chemically identical to one already written, and param (optional) = defines how “identical” is determined (if you omit [param], it uses InChI automatically, which is usually fine). This is the file that will be formatted for screening in Basic Protocol 2.

## Basic Protocol 2 ENSEMBLE‐BASED VIRTUAL SCREENING OF LOW‐MOLECULAR‐WEIGHT COMPOUNDS AGAINST RNA TARGETS

This protocol details the process of performing EBVS of small molecules against RNA targets using multiple conformers derived from FARFAR2 and docking with ICM. This approach accounts for RNA structural heterogeneity by averaging docking scores across conformational ensembles. Unlike the receptor preparation steps, the screening and data analysis are done in the command line.

### Materials

#### Hardware

Platforms (operating systems running on specific chip architecture); full list of supported platforms available at https://www.molsoft.com/products.html

##### Software

ICM version 3.9, Molsoft (https://www.molsoft.com/index.html), as docking software (here used for target and ligand preparation)
Python (https://www.python.org/downloads/) for data analysis
ChemAxon software version 19.21.0 (https://www.certara.com/chemaxon/), with Marvin tools used for creating new filtered .sdf libraries

##### Files

Ligand libraries from Basic Protocol 1
Receptor files from Basic Protocol 1
Scripts available on GitHub at https://github.com/Ken‐Lab‐ScrippsResearch/Curr‐Protoc‐2026‐SARSCoV2
Docking_Setup.icm
Ligand_Setup.icm
MakeHitList.icm
Run_Docking.csh
Docking_Processing.py

#### File preparation

1 Create a folder that contains all the receptor files and the ligand library file generated in Basic Protocol 1 as well as the .icm scripts.
2 Modify the “Docking_Setup.icm” file to include the names of the receptors of interest.
  This script should contain the following lines of code for each of the receptors you are docking in the ensemble. Shown here are the commands needed for one receptor, where you would replace “Receptor_1” with your receptor name (the prefix defined in the docking setup step, namely, Basic Protocol 1, step 6b, that is now the prefix of all your receptor map files). This needs to be repeated for all receptors in your ensemble.


dockUpdateGlobals "Receptor_1" ‐1
if(no & currentDockProj.l_readyReceptor) dockDisplayMol "Receptor_1" 0, ‐1
dockUpdateGlobals currentDockProj.data[1] ‐2
dockTableValue currentDockProj.data[1] "l_sampleRacemic" "logical" String(no)
dockTableValue currentDockProj.data[1] "l_dbondCisTrans" "logical" String(no)
dockTableValue currentDockProj.data[1] "l_softLigand" "logical" String(yes)
dockTableValue currentDockProj.data[1] "i_ringFlexLevel" "integer" String(2)
dockTableValue currentDockProj.data[1] "l_neutralAcidsMol" "logical" String(no)
dockTableValue currentDockProj.data[1] "s_chargeGroups" "string" "none"
if(currentDockProj.l_readyXray) dockTableValue currentDockProj.data[1] "r_densityWeight" "real" String($currentDockProj.data[49])
dockTableValue currentDockProj.data[1] "l_fixOmegas" "logical" String(no)
dockUpdateGlobals currentDockProj.data[1] ‐1

3 Modify the “Ligand_Setup.icm” file to include the names of the receptors, the name of the library, and the file path to the folder with the library and receptor files.
  This script should contain the following lines of code for each of the receptors you are docking in the ensemble. Shown here are the commands needed for one receptor, where you would replace “Receptor_1” with your receptor name, “Library.sdf” with your library name, and “Filepath” with the correct filepath to your working directory. This needs to be repeated for all receptors in your ensemble.


dockUpdateGlobals "Receptor_1" ‐1
if(no & currentDockProj.l_readyReceptor) dockDisplayMol "Receptor_1" 0, ‐1
currentDockProj.data[2:9] = {"dock3DbScanSetup"}//String(yes)//String(no)//"auto"// "mol" // "/Filepath/Library.sdf" // "yes" //String(yes)
dock3DbScanSetup currentDockProj.data[1] no yes "default"

4 Modify the “MakeHitList.icm” file to include the names of the receptors and the name of the library accordingly.
  This script should contain the following lines of code for each of the receptors you are docking in the ensemble. Shown here are the commands needed for one receptor, where you would replace “Receptor_1” with your receptor name and “Library” with your library name. This will be the prefix generated by the docking process, which you will use to process the output files. This needs to be repeated for all receptors in your ensemble.


scanMakeHitList "Receptor_1" "Receptor_1_Library" Name(Name("Receptor_1_Library"), simple) 3 ==2 no 3 ==1 no ? 100: 0

#### Initial docking (Tier0)

5 Start the ICM module in the command line.
  The command for this may be different depending on what version of ICM you are running and how your licenses are configured.
6 Run the “Docking_Setup.icm” file.
7 Run the “Ligand_Setup.icm” file.
  This should create a new indexed library file with the same name as your original .sdf file but now with an .inx suffix. This new .inx file is what ICM will call on to run docking.
8 Submit the docking job either with the run command or by executing a script that sends the job to a computing cluster.
  An example script of how we submit docking jobs is included as “Run_Docking.csh”. The primary docking command is as shown here:


_dockScan Receptor_1 from=1 to=20 input=Library.inx ‐a thorough=1. >! "Receptor_1"_Library.ou

  In this command, replace “Receptor_1” with your receptor name, “Library” with your library name, and “to = 20” to “to = #”, where # = the number of molecules in your library. This command must be executed for every receptor. “Run_Docking.csh” configures this command to dock multiple receptors in series.
  Once docking is completed, each ligand in the library obtains one docking score per receptor, as well as the pose of the ligand (3D coordinates) corresponding to that score. This information will be generated in two types of output files: an .ob file that has the prefix of your receptor and library, including the score and pose information, and a .ou file that contains information about the docking run.
9 To extract the score information from the output files, run the “MakeHitList.icm” script.
  This will generate a .tab file for each receptor with the scores for all ligands in your library.
  Everything described here constitutes one docking replicate. For docking RNA with ICM, multiple docking replicates are prudent, as each run has a different starting seed, which can produce different results. For large libraries, the computing cost of multiple replicates can be quite high, so the most economical approach is to perform two replicates of the entire library and then a higher number of replicates for a smaller subset of compounds that pass a score cutoff for either of the two initial replicates.

#### Data analysis

10 Put all the .tab files created with the “MakeHitList.icm” script into a new folder with the “Docking_processing.py” script. Concatenate all .tab files for the library into one file.
11 Edit the “Docking_Processing.py” file as follows:
  a. On line 7, enter the number of receptors in the ensemble, as indicated by the comment in the script.
  b. On line 8, enter the number of molecules in the library, as indicated by the comment in the script.
  c. On line 11, enter the name of the concatenated .tab file in place of “Filename.tab”.
  d. On line 42, enter the name of the receptor prefix without the trailing numerals (e.g., “Receptor” instead of “Receptor_1_1”) where it says “Receptor.”
12 Execute the “Docking_Processing.py” script.
  The output is two .csv files, “All_Score.csv” and “Score_Final.csv”. “All_Score.csv” includes the scores for each ligand against each individual receptor. This is a compact version of the raw docking values. “Score_Final.csv” has the score for each ligand averaged over all receptors.
  The molecule's ensemble docking score listed in “Score_Final.csv” is derived by calculating the mean of the scores for each receptor within the ensemble. Averaging of the docking scores is performed in two different ways, both of which are present in different columns in the “Score_Final.csv” file:
  Average score across all receptors: Arithmetic Average (AA; A_i_ = docking score for receptor i)
$$\langle AA\rangle =\frac{1}{N}\sum_{i}A_{i}$$
  Weighted average scores: Boltzmann Average [BA; A_i_ = docking score for receptor i, E_i_ = energy of state i (in our case, this is 1000*A_i_), k_B_ = Boltzmann constant, T = temperature]
$$\langle BA\rangle =\frac{\sum_{i}A_{i}e^{-E_{i}/k_{B}T}}{\sum_{i}e^{-E_{i}/k_{B}T}}$$
13 Perform aggregation of replicates:
  a. Determine AA and BA (see step 12) for each replicate.
  b. Select the best score across the replicates as the final Tier0 score.
    Scores will be negative values. The lower the score, the higher the predicted binding affinity. Thus, the best score is the lowest, most negative value. The AA and BA lists for scores for all small molecules in your library constitute your Tier0 dataset.
14 Determine statistical cutoff (Tier1 filtering):
  a. Compute the mean (µ) and standard deviation (σ) of the distribution of scores for the entire library (Tier0 dataset) for both the AA and BA scores.
  b. Apply a cutoff of at least 3 × σ:
cutoffscore=μ−3σ
    Only scores lower than this cutoff should move to the next phase.
  c. Exclude scores > 0.
  d. Apply additional AA cutoff.
    For the AA datasets, an additional cutoff score of –35 was applied, meaning that if the cutoff score is higher than –35, still only move forward with compounds lower than –35. This value was established through the analysis of previous findings by the Al‐Hashimi laboratory, which demonstrated that authentic positive results seldom exceed –35 (Ganser et al., 2018).
  e. Combine AA and BA hits and remove duplicates.
    The list of compounds after the score cutoff filtering for both BA and AA datasets constitutes your Tier1 compound list. There will likely be compounds that pass the cutoff for both the AA and BA datasets. Keep a record of the AA and BA scores for each ligand in this Tier1 list, but for the purposes of redocking, you only need one copy of the ligand in the next library file you make for redocking this set.
15 Prepare a new .sdf library file of the Tier1 compounds.
  Depending on which chemoinformatics software you use, this may have different steps. We used MarvinView in the Chemaxon software suite to view our original library file. From this file, you can save as a new .sdf and specify a subset of the compounds to include by their “IX” value, which is the number that indicates their order in the list. Save only the IX values that represent the compounds in your Tier1 list to make a new Tier1_Library.sdf file.

## Basic Protocol 3 COMPARATIVE HIT PRIORITIZATION AND SELECTIVITY FILTERING FOR RNA‐BINDING SMALL MOLECULES

This protocol describes a multi‐stage selectivity filtering strategy designed to identify small molecules that bind preferentially and specifically to the selected RNA structural element. RNA‐binding small molecules are well known to exhibit high levels of nonspecific binding due to the repetitive and highly charged nature of RNA surfaces and nonspecific hydrophobic interactions. As a result, conventional VS approaches that rely solely on docking scores against a single target structure often yield compounds that bind broadly to many RNAs rather than selectively to the intended target (Taghavi et al., 2025). To address this limitation, the present workflow incorporates progressive filtering steps (Tier1 to Tier3) that explicitly evaluate binding selectivity across multiple RNA contexts.

Basic Protocols 1 and 2 outlined the basic steps of VS with a full library (Tier0) and, at the end, produced a Tier1 library based on score cutoff values for a small number of replicates. With this Tier1 library, compounds that passed initial score‐based filtering are redocked against their target RNA ensemble with increased sampling (multiple replicates), generating a more robust estimate of binding affinity. The same compounds are cross‐docked against decoy RNA element ensembles, e.g., HIV‐related RNA structures, which serve as alternatives for nonspecific RNA binding. Compounds are then evaluated for predicted affinity and selectivity by comparing ensemble‐averaged docking scores between target and decoy RNAs. Selectivity is assessed using a statistical criterion, where a compound must show stronger predicted binding to the target RNA than to decoy RNAs (difference > several standard deviations from the decoy score distribution). The compounds that pass this selectivity filter become the Tier2 library. This library is further evaluated through expanded ensemble comparisons, and their docking poses may be visually inspected to eliminate geometrically implausible binding modes. This stage refines the candidate set by combining quantitative scoring with structural plausibility. Finally, in Tier3, compounds are subjected to cross‐docking against multiple related RNA elements, including both the original target and additional structurally distinct RNAs from the same organism or system. This step enables the identification of compounds that are selective relative to decoy RNAs, as well as specific to a single RNA element within a broader panel. Such cross‐element selectivity is crucial for avoiding off‐target interactions within the transcriptome.

This multi‐tiered approach (Table 1) substantially increases the likelihood that computational hits will translate into experimentally validated, selective RNA‐binding molecules, addressing a central challenge in RNA‐targeted drug discovery.

**Table 1:** Overview of the Different Stages (Tiers) of the Virtual Screening Approach, Including Their Purpose

Stage	Purpose
Tier0	Scores for full initial library
Tier1	Score‐based cutoff, target redocking
Tier2	Target vs. decoy selectivity
Tier3	Cross‐RNA specificity refinement

### Materials

#### Hardware

Platforms (operating systems running on specific chip architecture); full list of supported platforms available at https://www.molsoft.com/products.html

##### Software

ICM version 3.9, Molsoft (https://www.molsoft.com/index.html), as docking software (here used for target and ligand preparation)
Python (https://www.python.org/downloads/) for data analysis
ChemAxon software version 19.21.0 (https://www.certara.com/chemaxon/), with Marvin tools used for creating new filtered .sdf libraries
PyMOL open GL software version 2.1 (https://www.pymol.org/) for visualization of structures

##### Files

Receptor files from Basic Protocol 1
Tier1 ligand libraries produced from Basic Protocol 2
Structure files (.pdb) for decoy ensembles
Here, we use HIV‐1 TAR ensemble available on GitHub at https://github.com/Ken‐Lab‐ScrippsResearch/Curr‐Protoc‐2026‐SARSCoV2
FARFAR2 RNA ensembles, available on GitHub at https://github.com/DasLab/FARFAR2‐SARS‐CoV‐2
Scripts available on GitHub at https://github.com/Ken‐Lab‐ScrippsResearch/Curr‐Protoc‐2026‐SARSCoV2
Docking_Setup.icm
Ligand_Setup.icm
MakeHitList.icm
Run_Docking.csh
Docking_Processing.py

#### Tier1: Redocking against target ensemble

1 Prepare the Tier1 library created at the end of Basic Protocol 2 by editing “Ligand_Setup.icm” to include the new library name, size, and filepath.
2 Use the same receptor files for the target ensemble as prepared previously.
3 Edit “MakeHitList.icm” to include the new library name.
4 Edit “Run_Docking.csh” or analogous script to execute docking with the new Tier1 library.
5 Load ICM and run “Docking_Setup.icm” and “Ligand_Setup.icm” as in Basic Protocol 2, steps 5 to 7.
6 Run docking (see Basic Protocol 2, step 8). Repeat 10×.
7 Analyze each replicate of Tier01 target redocking as outlined in Basic Protocol 2, steps 9 to 14, using “MakeHitList.icm” to generate score files and “Docking_Processing.py” (edited with new library names) to calculate final AA and BA scores.

#### Tier1: Cross‐docking against decoy ensembles

8 Prepare receptors for decoy ensemble(s) as was done for the target ensemble in Basic Protocol 1.
  We used a previously determined ensemble of HIV‐1 TAR, which is included in the Materials list.
9 Edit “Docking_Setup.icm”, “Ligand_Setup.icm”, “MakeHitList.icm”, and “Run_Docking.csh” to include the correct library name and the new receptor names.
10 Load ICM and run “Docking_Setup.icm” and “Ligand_Setup.icm” as in Basic Protocol 2, steps 5 to 7.
11 Run docking (see Basic Protocol 2, step 8). Repeat 10×.
12 Analyze each replicate of Tier1 decoy cross‐docking as outlined in Basic Protocol 2, steps 9 to 14, using “MakeHitList.icm” to generate score files and “Docking_Processing.py” (edited with new receptor and library names) to calculate final AA and BA scores.

#### Tier2: Selectivity cutoffs

13 Apply a selectivity filter that selects compounds that bind significantly better to the target RNA than to the decoy RNA(s). To do this, mark each individual compound as either pass or fail based on the following criterion:
$$cutoff\;PASS=\;\mu \left(Target\right)<\left(\mu \left(Decoy\right)-3\sigma_{Decoy}\right)$$
  Perform this step for both the AA and BA score lists.
  In Basic Protocol 2, the mean (µ) and standard deviation (σ) used in the score cutoff were statistics describing the entire dataset of library scores, in which every ligand has one single score (the lowest of any replicates that were performed). In this protocol, we are using these statistics differently. Here, we are talking about the mean (µ) and standard deviation (σ) for a single compound across many redocking replicates, and the resulting cutoff score will be different for every single compound depending on how it performs against the decoy ensemble. This is why we must use a pass/fail cutoff on a per‐molecule basis for this filter.
14 Exclude compounds failing the selected cutoff.
15 Use PyMOL to visually inspect the top‐scoring poses of the compounds that pass this selectivity cutoff to exclude compounds that are artificially achieving excellent scores with implausible orientations, steric clashes, and other issues.
16 Prepare a new .sdf library file of the Tier2 compounds as done in Basic Protocol 2, step 15.

#### Tier3: Cross‐element selectivity refinement

Apply this step, especially when using different but related RNA structural ensembles as the target input. In our initial publication, we focused on two RNA elements derived from the SARS‐CoV‐2 RNA genome: SL1 and PK. Docking results for Tier3 compounds particularly reflect the selectivity between these two target RNA inputs.

17 Prepare receptors for target‐related RNA structural ensembles, as done in Basic Protocol 1, steps 1 to 8.
18 Edit all docking and data analysis scripts with the new library and new receptor information, as done in Basic Protocol 2, steps 1 to 4 and steps 10 to 12.
19 Run docking of Tier2 compounds against target‐related receptors, as done in Basic Protocol 2, steps 5 to 9 (four replicates).
20 Calculate AA and BA for all replicates, as done in Basic Protocol 2, steps 10 to 12.
21 Compute mean (µ) and standard deviation (*σ*) of replicate scores per compound based on AA and BA per compound for target RNA(s), as done in Basic Protocol 2, step 13 (also see step 13 of Basic Protocol 3).
  Choose a cutoff that makes sense for your system to distinguish between your primary target and the related targets screened in this step. The stringency you choose will mostly depend on how many compounds you are able to purchase, and the number of compounds left at this stage. In our case we were able to purchase all Tier2 compounds, so this Tier3 filtering step was for post‐hoc analysis of cross‐docking efficacy after experimental testing.
22 Use PyMOL to visually inspect the top‐scoring poses of the compounds that pass this filter.
  It may be useful to compare the binding poses of these compounds to those that do not pass and determine if there are any useful patterns in the pockets chosen by these compounds and in the modes of binding. If you need to make choices about which compounds to purchase (see step 23), it may make sense to select compounds that cover a range of predicted binding pockets and shape complementarity.
23 Purchase compounds that have passed Tier3 and, if desired, compounds that did not pass beyond Tier2 to test the accuracy of cross‐docking selectivity.

## Basic Protocol 4 PREPARATION OF RNA SAMPLES FOR *IN VITRO* VALIDATION OF SMALL‐MOLECULE BINDING

This protocol outlines the preliminary steps, including DNA plasmid design, amplification, and processing, for preparing RNA *in vitro* using T7 polymerase (Guillerez et al., 2005), up to the preparation of the working stock solution. Depending on the planned NMR experiments, RNA samples can be prepared with either naturally abundant isotopes or isotopically enriched, or labeled, nucleotides. Using ^15^N‐labeled nucleotides (NTPs), for example, enables recording experiments such as 2D‐^1^H,^15^N TROSY. Depending on the available budget and the composition and length of the construct sequence, RNA can also be ordered from companies such as Dharmacon^TM^ – Horizon Discovery (https://horizondiscovery.com/), which provides ready‐to‐use, synthesized, and purified RNA samples. RNA quality should always be checked prior to actually conducting the experiments (check for impurities and secondary structure fold).

### Materials

Competent *Escherichia coli* strain DH5α cells (New England Biolabs, cat. no. C2987H)
Luria broth (LB) medium (see recipe)
LB agar plates (see recipe; with appropriate antibiotic depending on plasmid design)
LB medium (see recipe; make fresh) + antibiotic
Super broth (SB) medium (see recipe; make fresh) + antibiotic
Liquid nitrogen
Qiagen Plasmid Giga Kit or similar plasmid purification kit
Appropriate restriction enzyme (depending on plasmid design)
Phenol‐chloroform‐isoamyl alcohol (PCI) solution (Carl Roth, cat. no. A156.1)
Chloroform
3 M sodium acetate (NaOAc), pH 5.5 (see recipe)
Isopropanol
70% (v/v) ethanol
Deionized water (H_2_O; Milli‐Q^®^ system, Merck)
1.5% (w/v) agarose in 1× Tris/acetic acid/EDTA (TAE) buffer, pH 8.0 (diluted from 50× TAE buffer, pH 8.0; see recipe)
6× gel‐loading dye, purple (New England Biolabs, cat. no. N0552S)
1‐kb DNA ladder (Quick‐Load® Purple 1 kb DNA Ladder, New England Biolabs, cat. no. B7024S)
*In vitro* transcription (IVT) reagents (see Table 2)
Yeast inorganic pyrophosphatase (YIPP; New England Biolabs, cat. no. M2403L)
0.5 M ethylenediaminetetraacetic acid (EDTA), pH 8.0 (see recipe)
2‐propanol or 1:1 ethanol/acetone
Formamide (Sigma‐Aldrich, Merck, cat. no. 47671‐250ML‐F)
RNaseZap^TM^ (Invitrogen^TM^) or similar solution for RNase decontamination
Preparative denaturing polyacrylamide (PAA) gel electrophoresis (PAGE) gel solution (see recipe)
Running buffer: 1× Tris/borate/EDTA (TBE) buffer, pH 8.0 (diluted from 10× TBE buffer, pH 8.0; see recipe)
RNA loading buffer (optional; see recipe)
100% ethanol
Buffer A: 0.1 M triethylammonium acetate (TEAA), pH 6.1 (see recipe)
Buffer B: acetonitrile
2% (w/v) lithium perchlorate (LiClO_4_) in acetone
NMR screening buffer (see recipe)
Native PAGE gel solution (see recipe)
1× Tris/NaOAc (TA) buffer, pH 8.2 (diluted from 10× TA buffer, pH 8.2; see recipe)
1:10,000 dilution of GelRed™ (Biotium, cat. no. BOT‐41003)
Deuterium oxide (d_2_O; Deutero, cat. no. 00506‐10ml)

Plasmid design software
42°C water bath
37°C shaking incubator
37°C bacterial incubator
Microcentrifuge
Culture flasks
Centrifuge tubes
Standard tabletop centrifuge, 4°C and room temperature
Vortex
UV/VIS spectrophotometer
Analytical agarose gel electrophoresis system
50‐ml reaction tubes
Preparative midigel system (Bio‐Rad Laboratories)
Cut, unfolded small autoclave bags
TLC plate (silica gel 60 matrix)
UV lamp (λ = 254 nm)
Scalpel
20‐ml syringes
65°C water bath
Shaker (Orbi‐Shaker, Benchmark Scientific, or Multi Reax, Heidolph Scientific Products)
Vacuum filter units (tube top, 50 ml, 0.22‐µm pore size)
Preparative reversed‐phase high‐performance liquid chromatography (RP‐HPLC) system
C18 column (Kromasil RP 18, 10 × 250 mm)
Lyophilizer or freeze dryer
80°C heat block
Centrifugal concentrators with suitable molecular weight cut‐off membrane
Gel imaging system (Molecular Imager^®^ Gel Doc^TM^ XR+ Imager, Bio‐Rad)

Additional reagents and equipment for pouring PAGE gels and for 1D‐^1^H spectrum measurement (see Basic Protocol 5)

**Table 2:** IVT Reagents Needed to Prepare SARS‐CoV‐2 SL1 RNA in a 10‐ml Reaction, Along with Their Respective Concentrations

Component	Σ concentration	Final concentration	Stock concentration	Volume [µl]		
Tris/glutamate, pH 8.1 [mM]		200.0	1000.0	2000.0		
DTT [mM]		20.0	1000.0	200.0		
Spermidine [mM]		2.0	200.0	100.0	**NTPs**	
Mg(OAc)_2_ [mM]		40.0	500.0	800.0	Sequence count [#]	Percentage [%]
ATP*^a^* [mM]	**14**	2.5	100.0	247.8	20	0.18
CTP*^a^* [mM]		4.7	100.0	470.8	38	0.34
GTP*^a^* [mM]		4.3	100.0	433.6	35	0.31
UTP*^a^* [mM]		2.5	100.0	247.8	20	0.18
Linearized plasmid DNA [ng/µl]		50.0	4000.0	125.0	113	1.00
DMSO*^b^* [%]		/	/	/		
T7 polymerase*^c^* [µg/ml]		32.0	2400.0	133.3		
Deionized H_2_O				5241.7		

^*a*^ The ratios of the NTPs are adjusted to the target sequence composition. The NTPs are at natural abundance (n.a.) or are labeled.

^*b*^ DMSO is used if needed to reduce secondary structure in the DNA template, improving processivity of the T7 polymerase.

^*c*^ In‐house‐prepared T7 polymerase (P266L mutant).

#### Plasmid design, amplification, and priming

##### Design DNA templates

1 Using plasmid design software, incorporate target RNA sequences with a T7 promoter.
2 Introduce sequences into a suitable vector (e.g., pSP64 or HDV‐ribozyme‐encoding plasmid) via restriction sites (EcoRI/NcoI or EcoRI/HindIII). Include an appropriate and convenient antibiotic resistance cassette.
3 Order plasmid from GenScript (https://www.genscript.com/) or similar and handle according to the manufacturer's guidelines after arrival.
  The plasmid we use includes the T7 promotor, an ampicillin resistance cassette, HDV ribozyme, and the pSP64 vector (Promega).

###### Transformation

4 Thaw 50 µl stock of competent *E. coli* strain DH5α cells on ice.
5 Add 50 ng plasmid DNA from step 3 and incubate 15 min on ice.
  Steps including any bacterial cultures are handled within a sterile fume hood to avoid any cross‐contamination.
6 Heat‐shock at 42°C in a water bath for 30 sec and then return to ice 5 min.
7 Add 450 µl LB medium and incubate at 37°C and 120 rpm for 60 min.
8 Centrifuge cells for 3 min at 5000 × *g* at room temperature and then discard 400 µl supernatant. Use the remaining solution to resuspend the cells.
9 Plate 20 µl on LB agar plates (with appropriate antibiotic).
  We use ampicillin with a final concentration of 100 ng/µl.
10 Incubate plates upside down overnight at 37°C.
  To ensure sequence accuracy, the ordered plasmid can be cross‐checked with an external sequencing service of choice before or after transformation.

###### Bacterial culture and plasmid amplification

11 Inoculate single colony into 50 ml LB medium + antibiotic in a culture flask and incubate at 37°C and 120 rpm for 8 hr.
12 Transfer 10 ml starter culture into 1 L SB medium + antibiotic and incubate overnight at 37°C and 120 rpm.
13 Harvest cells using a suitable centrifuge tube, according to the size of the culture and the available centrifuge system, by centrifugation for 15 min at 6000 × *g*, 4°C. Discard supernatant and freeze pellet in liquid nitrogen and store at –80°C if purification and linearization of the plasmid DNA are not immediately performed.

###### Plasmid purification and linearization

14 Purify plasmid DNA using the Qiagen Plasmid Giga Kit or similar plasmid purification kit according to the manufacturer's instructions.
15 Digest 10 mg plasmid with appropriate restriction enzyme (e.g., HindIII) in 2.4 ml reaction volume according to the manufacturer's instructions. Incubate overnight at recommended temperature.
16 Perform PCI extraction to remove proteins:
  a. Add 1 volume PCI, mix via vortexing, and centrifuge 10 min at 10,000 × *g*.
  b. Transfer aqueous phase to a new tube and repeat PCI extraction.
  c. Extract once with 1 volume chloroform.
  d. Precipitate DNA: add 1/10 volume of 3 M NaOAc (pH 5.5), mix with 0.7 volume isopropanol, and incubate at –20°C for 1 hr.
  e. Centrifuge 30 min at 10,000 × *g*, discard supernatant and wash pellet with 70% ethanol, and air‐dry pelleted DNA for 5 to 10 min.
  f. Dissolve DNA in deionized H_2_O and quantify concentration by UV/VIS spectrophotometry at 260 nm.
  g. Confirm integrity by agarose gel electrophoresis in 1.5% agarose in 1× TAE buffer (pH 8.0), with 100 ng/µl DNA in 1× gel‐loading dye, using an analytical agarose gel electrophoresis system. Include a 1‐kb DNA ladder.
    Samples checked on the agarose gel should represent DNA before digestion, linearized DNA, and DNA after PCI extraction.

###### T7 polymerase–based in vitro transcription of RNA

17 Mix the listed components in Table 2 according to their order of mention in a 50‐ml reaction tube. Gently vortex before adding T7 polymerase.
  The amount of added NTPs depends on their sequence abundance. The presence of dimethyl sulfoxide (DMSO) might increase the efficiency of T7 polymerase but should be checked on a smaller scale beforehand. Transcription optimization on a smaller scale (10 µl is sufficient) with variations in conditions such as the concentration of the template DNA applied (typically 50 to 200 ng/µl) and supplements like dithiothreitol (DTT) and magnesium acetate (Mg(OAc)_2_) is a prerequisite for preparative IVT.
  Water should always be added first to prevent other supplements from precipitating due to excessively high local concentrations. T7 polymerase should always be the last component added, allowing for gentle vortexing of the previously combined IVT components before the addition of the enzyme.
  T7 polymerase was prepared as previously described by the research group of Dreyfus (Guillerez et al., 2005). We chose the P266L T7 polymerase mutant because of its increased ability to stabilize the transcription initiation complex and its proven reduction of abortive RNA products. This mutant utilizes the standard T7 promoter sequence (5’‐TAATACGACTCACTATA‐3’), and the target RNA construct is preferably designed to begin with at least two guanosine residues at the 5’ terminus to optimize transcription initiation.
18 Incubate the reaction at 37°C with mild shaking (60 rpm) for ∼6 hr.
  The quality and efficiency of the transcription should always be checked via analytical denaturing PAGE in parallel to the actual transcription.
19 Add YIPP according to the manufacturer's recommendation, or to a final concentration of 9.6 µg/µl if prepared in‐house, after 1 to 2 hr if turbidity becomes visible.
  This prevents toxic pyrophosphate accumulation.
20 Terminate the transcription after incubation as follows:
  a. Centrifuge IVT mixture for 5 min at 10,000 × *g* at room temperature.
  b. Transfer the supernatant to a fresh 50‐ml reaction tube.
  c. Add 0.5 M EDTA (pH 8.0) to 80 mM and 0.1 volumes of 3 M NaOAc (pH 5.5), followed by the addition of 1 volume of ice‐cold 2‐propanol (or 1:1 ethanol/acetone for <30 nt) to precipitate the RNA.
  d. Store the suspension at –20°C overnight.
21 Centrifuge the ice‐cold suspension for 1 hr at 10,000 × *g*, 4°C.
22 Discard the supernatant and wash the pellet with 5 ml of 70% ethanol. Centrifuge 15 min at 10,000 × *g*, 4°C.
23 Discard the supernatant and air‐dry the pellet for 5 to 10 min.
24 Resuspend the RNA pellet in an appropriate volume of deionized H_2_O (1 to 2 ml). Add an equal volume of formamide to the resuspended RNA. Store at –20°C or proceed with direct PAGE purification, storing on ice before loading the sample onto the gel.

#### RNA purification via PAGE and HPLC

##### Denaturing PAGE purification

25 Prepare the preparative midigel system by cleaning with RNaseZap^TM^ or a similar solution for RNase decontamination and pour the preparative denaturing PAGE gel.
  A final PAA concentration of 12% (w/v) is well suited for RNAs like SL1 (29 nucleotides, HDV ribozyme).
26 Assemble the midigel system and fill the gel chamber with the appropriate volume of running buffer (1× TBE buffer, pH 8.0).
27 Wash the gel pocket with 1× TBE buffer to remove any residual PAA.
28 Load the sample from step 24 stepwise. If available, apply RNA loading buffer to help in the estimation of the appropriate gel running time.
  Check the sample volume that can be loaded into the gel pockets, which differs between midigel systems and gel sizes.
29 Run the gel under established conditions.
  For a 12% PAA gel using a vertical midigel electrophoresis system (Bio‐Rad Laboratories), a 29‐nucleotide RNA containing a 3’‐terminal HDV ribozyme is typically resolved after 4 hr at 240 V.
30 After the run is complete, transfer the glass plates to a freshly disinfected working bench. Carefully remove the upper glass plate and place a cut, unfolded small autoclave bag on top of the gel. Invert the assembly so that the autoclave bag faces the bench and the former lower glass plate is now on top. Remove the remaining glass plate and cover the gel with the other side of the autoclave bag.
31 Place the covered gel onto a TLC plate and visualize the separated RNA bands in a dark room using a UV lamp (top illumination).
32 Use a pen to highlight the RNA band of interest, drawing directly on the autoclave bag.
*IMPORTANT NOTE*: The longer you expose the RNA to UV light, the greater the risk of RNA damage. Be fast but precise while highlighting the RNA band of interest during UV exposure. Wear appropriate PPE while doing so.
33 Perform RNA elution (crush‐and‐soak method):
  a. Open the autoclave bag on the side opposite the outlined RNA band and excise the region containing the target RNA using a scalpel. Cut the gel slice into small pieces.
  b. Transfer gel slices into a 20‐ml syringe placed inside a 50‐ml reaction tube containing 20 ml of 0.3 M NaOAc (pH 5.5).
  c. Mechanically disrupt gel pieces by repeatedly pushing them through the syringe to increase surface area.
  d. Freeze the suspension at –80°C for 30 min and then incubate in a 65°C water bath for 15 min.
  e. Elute RNA by shaking at ∼1300 rpm at room temperature overnight.
  f. Filter the supernatant to remove gel debris using vacuum filter unit.
  g. Repeat elution with fresh 10 ml of 0.3 M NaOAc buffer for 2 hr and combine eluates.
  h. Precipitate RNA by adding 4 volumes of 100% ethanol and incubating at –20°C for ≥2 hr or overnight.
34 Centrifuge 1 hr at 10,000 × *g*, 4°C.
35 Discard the supernatant and wash the pellet with 5 ml of 70% ethanol. Centrifuge 15 min at 10,000 × *g*, 4°C.
36 Discard the supernatant and air‐dry the pellet for 5 to 10 min.
37 Resuspend the RNA pellet in an appropriate volume of deionized H_2_O (∼0.5 ml).
  Use a UV/Vis spectrophotometer to check the salt content of the sample by evaluating the A260/A230 ratio. Elevated salt contamination may interfere with the efficiency of subsequent RP‐HPLC purification. Desalting the RNA sample may be necessary before the follow‐up purification.

###### RP‐HPLC of RNA

38 Further purify RNA using a preparative RP‐HPLC system with a C18 column (Kromasil RP 18, 10 × 250 mm).
39 Equilibrate column with buffer A (0.1 M triethylammonium acetate, pH 6.1).
40 Elute RNA using a 0% to 40% acetonitrile gradient (buffer B) over 40 min at 2.5 ml/min.
41 Collect fractions and analyze by denaturing PAGE to identify pure RNA.
42 Lyophilize or freeze‐dry RNA‐containing fractions to remove volatile buffers.
43 Resuspend RNA in 500 µl deionized H_2_O.
44 Precipitate RNA by adding 5 volumes of 2% LiClO_4_ in acetone and incubate at –20°C overnight.
45 Centrifuge 1 hr at 10,000 × *g*, 4°C. Discard the supernatant and repeat precipitation if necessary to ensure complete salt exchange.
46 Resuspend purified RNA in ∼500 µl deionized H_2_O.and store at –20°C until further use.

###### Quality control of freshly synthesized RNA (native PAGE and NMR)

47 Determine the concentration of the dissolved RNA via UV/VIS spectroscopy. Transfer a sample including 500 pmol RNA into a separation reaction tube and store on ice.
48 Perform a refolding of the RNA by classical snap cooling: heat 30 sec at 80°C using a heat block, followed by immediate cooling on ice for 5 to 10 min.
  The presented follow‐up analytical steps reveal if this method is suitable for the chosen construct. Alternative methods could include dilution of the RNA stock prior to snap cooling or stepwise cooling after heating.
49 Replace water with the actual NMR screening buffer and concentrate after folding of the RNA using a centrifugal concentrator with a suitable molecular weight cut‐off membrane. Centrifuge according to the manufacturer's recommendations.
*IMPORTANT NOTE*: Higher concentrations of RNA stocks increase the likelihood of intermolecular interactions, such as dimer formation or other off‐target secondary structure formation. For instance, the SARS‐CoV‐2 SL1 RNA construct exhibits detectable dimerization at 1.2 mM.
50 Determine the RNA concentration via UV/VIS spectroscopy.
51 Check folding homogeneity via native PAGE (in 1× TA buffer, pH 8.2; 500 pmol RNA per lane in 10% glycerol). Include the sample that was set aside in step 47 as a control to indicate unfolded RNA. Image native PAGE via UV shadowing, as described in step 31. Additionally, stain the gel with a 1:10,000 dilution of GelRed™ and visualize with a gel imaging system according to the manufacturer’s protocol.
  A total of 500 pmol RNA is loaded to replicate the RNA concentrations commonly used in NMR studies, ensuring consistency with structural measurements.
52 Prepare ∼100 µM RNA in 5% D_2_O and measure a jump‐return (jr) 1D‐^1^H spectrum as in Basic Protocol 5 to detect contaminants (e.g., acrylamide) and to check the secondary structure fold. If impurities are detected, repeat HPLC purification.

## Basic Protocol 5 NMR‐BASED *IN VITRO* SCREENING OF RNA–SMALL MOLECULE INTERACTIONS

Basic Protocol 5 outlines the use of NMR to screen a large set of compounds, how to classify them as actual hits, and how to perform follow‐up binding‐site mapping via NMR (Binas et al., 2021; Sreeramulu et al., 2021; Toews, Ceylan, et al., 2025; Toews, Donà, et al., 2025). Our method depends on the use of a spectrometer that is equipped with an automated system enabling the measurement of racks including 96 individual samples at once. We use the SampleJet^TM^ setup from Bruker, which offers storage for up to 480 tubes in total (96 positions per rack). The setup also allows for temperature pre‐equilibration. It also features spinner‐free operation. The setup supports tubes of 5 mm, 3 mm, 1.7 mm, and 1 mm in diameter and features automated sample insertion, barcode tracking, and integration with TopSpin^TM^ and IconNMR^TM^ software. The setup allows for the rapid initial screening of natural‐abundance RNA samples within an experimental setup, including three proton‐ and ligand‐detected experiments. A detailed protocol describing this kind of fragment‐based screening handling even larger fragment libraries was recently published by our group (Berg et al., 2021). The experimental procedure here applies three individual ligand‐detected experiments for initial hit identification (Table 3). In follow‐up experiments, binding affinities of RNA‐ligand complexes can be determined (not presented here). Additionally, binding‐site mapping may be performed using proton‐detected NMR experiments or, if isotopically labeled RNA is available, using ^13^C‐ or ^15^N‐detected NMR techniques.

**Table 3:** Experiments Used to Screen RNA‐Ligand Interactions via NMR

NMR experiment	Sequence information	Experiment‐specific parameter settings	Measurement [min]
1D‐^1^H	1D‐^1^H with SOGGY water suppression	Excitation sculpting using a composite 180° pulse; 32‐128 scans	4.5
waterLOGSY (^1^H)	waterLOGSY with SOGGY water suppression	Standard waterLOGSY sequence; mixing time = 1.85 ms; 256‐512 scans	27
*T_2_*‐CPMG (^1^H)	*T_2_* relaxation (pseudo‐2D, CPMG with spinlock field of 6.25 kHz, 5 & 100 ms, temperature‐compensated)	Standard CPMG sequence; 64‐128 scans	13

### Materials

RNA (see Basic Protocol 4) and ligand (see Support Protocol) working stocks
NMR screening buffer (see recipe)
5% (v/v) d_6_‐DMSO (*d_6_*‐Deutero, cat. no. 00905‐10ml)
Deuter

1.7‐ or 3‐mm NMR tubes
96‐tube racks (1.7‐mm diameter, Bruker, cat. no. Z106462, 3‐mm diameter, Bruker, cat. no. Z112272)
1.5‐ml reaction tubes
Vortex
Mini centrifuge
Extra‐long pipet tips
Barcode scanner connected to computer (optional)
POM balls (Bruker, cat. no. Z147554)
Benchtop centrifuge with rack insert/adapter
NMR spectrometer (600 MHz, Bruker AV NEO, cryogenic probe, SampleJet^TM^ autosampler)
TopSpin^TM^ equipped with IconNMR^TM^ software (automated measurements and analysis)
PyMOL open GL software version 2.1 (https://www.pymol.org/)

#### Screening sample preparation (RNA‐ligand and RNA/ligand‐only samples)

1 For 1.7‐mm NMR tubes, plan to use 40 µl total volume; for 3‐mm tubes, plan to use 180 µl total volume. Place the appropriate number of NMR tubes in a 96‐tube rack.
2 Prepare NMR samples in 1.5‐ml reaction tubes, containing 10 µM RNA only, 200 µM ligand only, or 10 µM RNA + 200 µM ligand at a final [RNA]/[ligand] ratio of 1:20 in NMR screening buffer. Also supplement the final NMR samples with 5% *d_6_*‐DMSO.
  To ensure sample integrity, always refold your RNA after storage at –20°C, as presented in Basic Protocol 4, step 48.
  Be aware that based on the results of the QC performed in the Support Protocol, some compounds might require more than 5% d_6_‐DMSO for sufficient solubility of the compound. Please check beforehand (also via NMR and additional native and denaturing PAGE) if the target RNA tolerates higher DMSO concentrations.
3 Carefully vortex the reaction tubes.
4 Briefly centrifuge the tubes in a mini centrifuge to ensure liquid is not remaining in the lids of the reaction tubes after vortexing.
5 Transfer the samples into the NMR tubes from step 1 by stepwise pipetting (2 × 20 µl) using extra‐long pipet tips. After the transfer of the first 20 µl, if needed, flick tubes by hand to ensure free space for the remaining 20 µl.
6 If available, scan the barcodes of the NMR lids using a barcode scanner connected to a computer.
  Prepare a Microsoft Excel table with the layout of a 96‐tube rack in advance.
  This is needed for proper documentation of the samples, as manual labeling of the tubes in a high‐throughput approach will be cumbersome. The SampleJet^TM^ setup also scans the barcode before every measurement, helping in relocation of the sample's original position in case of measurement failure.
7 Close the lids of the NMR tubes using POM balls.
8 After preparing all NMR samples, use a benchtop centrifuge with a rack insert/adapter to briefly spin the racks including the NMR samples.
  This will ensure that all the sample liquid is collected at the bottom of the tube and helps to exclude any air bubbles occurring after pipetting and flicking of the sample.

#### General acquisition setup

9 Load samples into a SampleJet^TM^ autosampler of the NMR spectrometer and control measurements using TopSpin^TM^ equipped with IconNMR^TM^ software for automated acquisition.
  The SampleJet^TM^ system includes a sample storage unit allowing manual setting of the storage temperature for all the samples that are on hold. We use 4°C as the storage temperature; samples will be preheated by the system before actual measurements.
10 Set measurement temperature to 298 K unless RNA stability requires adjustment.
11 Perform automatic tuning and matching, followed by gradient shimming on the H_2_O signal (e.g., using a ^1^H‐based shimming routine).
12 Suppress residual water signal in all experiments using the solvent‐optimized double gradient spectroscopy (SOGGY) pulse sequence (Nguyen et al., 2007).
  SOGGY is preferred over excitation sculpting for screening experiments, as it provides improved robustness, particularly in the presence of air bubbles in the NMR tube and reduced sample filling height (common in 1.7‐ or 3‐mm tubes).

#### 1D‐^1^H experiment

13 Record a 1D‐^1^H experiment for the detection of CSPs and/or line broadening. Use 32 to 128 scans depending on signal intensity.
  All the experiments must be recorded for the RNA‐only, ligand‐only, and RNA‐ligand samples to enable direct comparison. To ensure sample quality, we recommend a blank measurement of the buffer in the absence of any RNA or ligand.
  All spectra should be referenced to an appropriate internal standard. We use the ^1^H signal of d_6_‐DMSO, which appears as a quintet at 2.68 ppm in our aqueous buffer.
14 Evaluate spectra for CSPs and line shape changes (line broadening).
15 Apply the following formula for the determination of CSPs:
$$CSP\;\left[\Delta \delta_{obs}\right]=\;\left|\delta_{bound}-\;\delta_{free}\right|$$

#### waterLOGSY experiment

16 Acquire waterLOGSY spectra using the SOGGY water suppression sequence (Dalvit, 1998; Dalvit et al., 2001).
17 Use 256 to 512 scans to ensure sufficient sensitivity.
18 Phase spectra such that positive signals indicate bound ligands and negative signals indicate unbound ligands.
19 Compare ligand‐only‐ and RNA‐only‐containing samples to identify binding‐induced signal inversion or intensity changes.
20 Use the following formula to determine the waterLOGSY factor:
$$wLOGSY\;factor=\left|\frac{\left(I_{Target}-(I_{Reference}\right)}{I_{Reference}}\right|$$
  This factor is the difference between the absolute peak intensities of the ligand in the presence (I_Target_) and absence (I_Reference_) of the RNA target, normalized to the reference intensity (I_Reference_). Ligand peaks to be analyzed need to be integrated using TopSpin^TM^.

#### T_2_ relaxation (CPMG) experiment

21 Acquire ^1^H‐*T_2_* relaxation data using the CPMG pulse sequence with SOGGY water suppression (Hajduk et al., 1997). Use 64 to 128 scans per experiment. Record spectra at two mixing times (e.g., 5 ms and 100 ms) with a CPMG pulse bandwidth of ∼6.25 kHz. Perform experiments with temperature compensation to ensure reproducibility.
22 Evaluate signal attenuation between mixing times using the following formula:
$$T_{2}\;\mathrm{reduction}=\left[1-\frac{\frac{I_{\mathrm{Target}}\;\left(100\;\mathrm{ms}\right)}{I_{\mathrm{Reference}}\;\left(100\;\mathrm{ms}\right)}}{\frac{I_{\mathrm{Target}}\;\left(5\;\mathrm{ms}\right)}{I_{\mathrm{Reference}}\;\left(5\;\mathrm{ms}\right)}}\right]\;\times \;100$$
  Binding is indicated by reduced signal intensity and line broadening, reflecting an increased rotational correlation time for the RNA‐ligand complex. Ligand peaks to be analyzed need to be integrated using TopSpin^TM^.

#### Binding‐site mapping via NMR

23 Proceed with binding‐site mapping if an assignment of the target RNA(s) is available.
  Different from the initial screening experiments, this approach is based on the detection of RNA resonances. For a better resolution, higher RNA concentrations are desired. This procedure does not require the entire set of ligands. Proceed with the most promising ones (see Basic Protocol 6 to determine which compounds are promising).
  2D‐^1^H,^1^H‐total correlation spectroscopy (TOCSY) experiments are especially useful when the predicted binding pocket includes several pyrimidine residues (C and U). This method is less cost efficient, as it functions as a proton‐detected NMR technique. In addition to TOCSY experiments, 2D‐^1^H,^1^H‐nuclear Overhauser effect (NOE) spectroscopy (NOESY) can be performed as a complementary experiment, particularly if the aim is to determine ligand‐RNA (intermolecular) NOEs for structure calculations for the complex.
24 Employ the DIPSI2 sequence (Bruker pulse program: dipsi2esfbgpph) with a mixing time of 30 ms (Braunschweiler & Ernst, 1983; Furrer et al., 2004).
25 Achieve water suppression in the direct dimension using excitation sculpting.
26 Record TOCSY spectra for RNA alone and RNA in the presence of ligand.
27 Determine CSPs for H5‐H6 cross peaks upon ligand binding using the Euclidean formula:
$$CSP\;\left[\Delta \delta_{HH}\right]=\sqrt{{\left(\Delta \delta_{H5}\right)}^{2}+{\left(\Delta \delta_{H6}\right)}^{2}}$$
28 Report CSP values for each residue to quantitatively characterize ligand binding.
29 Plot the CSPs onto the 3D structure in PyMOL, if available.
  This will aid in comprehending the effect of ligand binding on the RNA structure and can be utilized as cross‐validation of the docking poses obtained with Basic Protocols 1 to 3.

## PREPARATION OF LIGAND STOCKS AND NMR‐BASED QUALITY CONTROL

To ensure compound quality, all ligands are first checked by recording a single ^1^H spectrum to verify purity and solubility. For small libraries, individual measurements are sufficient. For larger libraries, ligands with clearly distinct NMR signals can be combined into mixtures to speed up QC while still detecting impurities or solubility issues. Only compounds passing these checks are advanced to RNA‐binding experiments.

### Additional Materials (also see Basic Protocol 5)

Purchased small molecules (solid)
*d_6_*‐DMSO (90%) / D_2_O (10%) (*d_6_*‐DMSO, Deutero, cat. no. 009025‐10ml; D_2_O, Deutero, cat. no. 00506‐10ml)
*d_6_*‐DMSO (Deutero, cat. no. 009025‐10ml)
40 mM sodium 4,4‐dimethyl‐4‐silapentane‐1‐sulfonate (DSS) in D_2_O

Suitable reaction tubes

#### Preparation of ligand stock solutions

1 Dissolve purchased small molecules in *d_6_*‐DMSO (90%) / D_2_O (10%) to prepare concentrated stock solutions (e.g., 10 to 50 mM). Use suitable reaction tubes for the amount of available compound.
2 Store ligand stocks at –20°C and avoid repeated freeze‐thaw cycles.

#### Quality control of ligands

3 Thaw the ligand stocks.
4 Prepare 1 mM ligand solutions in NMR screening buffer.
5 Include 5% *d_6_*‐DMSO and 10 µM DSS (diluted from 40 mM DSS in D_2_O) as internal chemical shift reference.
6 Record ^1^H spectra at 298 K to assess purity and solubility prior to RNA binding experiments.
7 Discard compounds from the final library that show impurities or reduced solubility.
  For very small libraries at the early stages of drug discovery, compounds can optionally be measured in the presence of slightly higher DMSO concentrations [up to 15% (v/v)] to improve solubility of poorly soluble ligands, without further reducing the effective library size.

## Basic Protocol 6 CHARACTERIZATION AND PRIORITIZATION OF VALIDATED RNA‐BINDING HITS

After NMR‐based screening (Basic Protocol 5), compounds for which interaction with target RNA constructs has been documented experimentally undergo further characterization to classify binding specificity, evaluate molecular features, and prioritize hits for downstream validation. This process combines NMR binding data with computational analyses of molecular descriptors and structural properties. The goal is to identify selective ligands, understand how they differ from non‐binding molecules, and gain molecular insights to guide future RNA‐targeted drug discovery.

Before undergoing detailed molecular characterization, compounds must first be classified as hits based on predefined quantitative NMR thresholds. These thresholds ensure reproducibility, minimize false positives, and capture meaningful binding events. The thresholds are selected based on prior experimental experience. Significant CSPs indicate direct interactions with RNA. *T_2_* reductions reflect complex formation and slowed molecular tumbling. Positive waterLOGSY signals confirm ligand binding via inversion of signal intensity relative to free ligand.

### Materials

Ligand hits validated via NMR (SMILES codes)
NMR results (CSPs, waterLOGSY factors, *T_2_* relaxation reductions; see steps 15, 20, and 22 of Basic Protocol 5)
Microsoft Excel
Python (3.x environment), with packages:
  RDKit (v2024.03.5; https://www.rdkit.org/)
  Pandas (v2.2.2)
  SciPy (v1.14.0)
  scikit‐learn (v1.6.1)
  umap‐learn (v0.5.7)
  Matplotlib (v3.9.1)
  Seaborn (v0.13.2)
Jupyter Notebook (v7.0.8, https://docs.jupyter.org/en/)
Anaconda distribution (v24.5.0, https://anaconda.com/)
DataWarrior (v06.04.02, https://openmolecules.org/datawarrior/), for chemical similarity analysis

#### Hit classification

1 Set suitable criteria for classifying ligand hits validated via NMR based on previously determined NMR results (CSPs, waterLOGSY factors, and *T_2_* relaxation reductions; see steps 15, 20, and 22 of Basic Protocol 5).
  According to our experimental experience, compounds can be classified as hits if the following criteria are met:
*a. CSP of ≥3 Hz*.
*b. WaterLOGSY factor of ≥1*.
*c. T_2_ relaxation reduction of ≥30%*.
2 Classify ligands that meet at least two of the criteria as hits. If feasible, conduct the analysis on several ligand resonances.
  Application of multiple orthogonal criteria will help to avoid false positives in NMR screening (nonspecific binders).
3 Document all hit compounds in a summary table, including CSP values, *T_2_* changes, and waterLOGSY scores.
4 Rank the results of the three individual NMR experiments within the overall data series of all measured compounds (Table 4):
  a. Determine the compounds with the highest CSP, waterLOGSY factor, and *T_2_* reduction using the Microsoft Excel rank function.
  b. Use the rank position to calculate the sum of the ranks throughout the three experiments.
  c. Determine the rank of the previously calculated sum by applying the Microsoft Excel rank function.
    In general, a higher total of individual ranks corresponds to a lower overall rank, meaning the hit is less strong.

**Table 4:** Example Layout of a Table Used for Hit Ranking

				Hit ranking				
Compound	CSP [Hz]	waterLOGSY factor	*T_2_* reduction [%]	Highest CSP	Highest waterLOGSY	Highest *T_2_*	Sum	Rank of sum
**x.1**	5.52	0.00	67	7	5	6	18	**4**
**x.2**	8.19	0.69	90	5	3	1	9	**1**
**x.3**	9.18	0.47	85	4	4	4	12	**3**
**x.4**	11.22	0.00	87	1	5	3	9	**1**

#### Optional: Molecular characterization and structural analysis

5 Process all data in Python using RDKit within a Jupyter Notebook, running on Anaconda distribution. Use Pandas library for data processing and data management. Apply the spicy.stats module of the SciPy library for statistical evaluations. For dimensionality reduction techniques, principal component analysis (PCA) can be performed with scikit‐learn, and uniform manifold approximation and projection (UMAP) can be performed with the umap‐learn package. Matplotlib and Seaborn are finally used for creating charts and heatmaps.
6 Prepare ligand structures computationally following a standard protocol (Getting Started with the RDKit in Python, https://www.rdkit.org/docs/GettingStartedInPython.html):
  a. Start from 2D representations.
  b. Add explicit hydrogens.
  c. Generate up to 200 conformers per ligand.
  d. Minimize energy using MMFF94s force field.
7 Calculate physicochemical descriptors relevant for RNA recognition:
  a. Molecular weight (MolWt).
  b. LogP.
  c. Topological polar surface area (TPSA).
  d. Hydrogen bond donors/acceptors (NumHDonors, NumHAcceptors).
  e. Rotatable bonds (NumRotatableBonds).
8 Compare descriptor distributions between binding categories using one‐way ANOVA, significance p < 0.05.
9 Generate Morgan fingerprints (ECFP4) with radius = 2, length = 2048 bits.
10 Identify substructures enriched in binders as follows:
  a. Present in >50% of a binding category (SL1 or PK).
  b. Present in <10% of non‐binding compounds.
11 Highlight motifs associated with selectivity and prioritize for future ligand design.
12 Use DataWarrior to calculate pairwise similarities (chemical similarity analysis):
  a. Prepare a DataWarrior table/sheet, including all compound properties to compare.
    Here, we present an example of comparing the structural scaffold of the hits and non‐hits in general.
  b. Apply SkelSpheres descriptor for scaffold topology.
  c. Compute Tanimoto similarity coefficients.
  d. Perform hierarchical clustering to visualize chemotype relationships.
13 Combine NMR‐based classification, physicochemical descriptors, fingerprints, and similarity clustering to prioritize hits for downstream assays and further structural characterization.

## REAGENTS AND SOLUTIONS

### Buffer A

0.1 M TEAA
Adjust pH to 6.1 using acetic acid, as TEAA is formed by titration of triethylamine with acetic acid
Store ≤1 month at 4°C

### EDTA (pH 8.0), 0.5 M

0.5 M EDTA in deionized H_2_O
Adjust pH to 8.0 using NaOH, as EDTA only fully dissolves upon deprotonation at alkaline pH
Store ≤1 year at 4°C

### LB agar plates

1.5% (w/v) agar
100 µg/ml ampicillin in LB medium (see recipe; make fresh)
Pour into Petri plates and let solidify
Store ≤1 month at 4°C
Pre‐warm to room temperature before use

### LB medium

1% (w/v) tryptone
0.5% (w/v) yeast
1% (w/v) NaCl
Transfer into sterile 5‐L culture flasks
Autoclave
Prepare immediately before use
Add appropriate antibiotic before inoculation
  We use 100 µg/ml ampicillin.

### NaOAc (pH 5.5), 3 M

3 M NaOAc in deionized H_2_O
Adjust pH to 5.5 using glacial acetic acid (to lower pH) or NaOH (to increase pH), with final fine‐tuning using acetic acid
Store ≤1 year at room temperature

### Native PAGE gel solution

1× TA buffer, pH 8.2 (diluted from 10× TA buffer, pH 8.2; see recipe)
Acrylamide/bis‐solution 30 (Carl Roth, cat. no. A124.2; concentration according to experimental needs)
Store ≤1 year at 4°C
Add 0.1% (v/v) ammonium persulfate (APS) and 0.1% (v/v) *N,N,N′,N′*‐tetramethylethylenediamine (TEMED; Carl Roth, cat. no. 2367.2) immediately before use

### NMR screening buffer

25 mM potassium phosphate (KPi), pH 6.2
50 mM potassium chloride (KCl)
Store ≤2 months at 4°C

### Preparative denaturing PAGE gel solution

8 M urea
1× TBE buffer, pH 8.0 (diluted from 10× TBE buffer, pH 8.0; see recipe)
Acrylamide/bis‐solution 30 (Carl Roth, cat. no. A124.2; concentration according to experimental needs, e.g., 12%)
Store ≤1 year at 4°C
Add 0.1% (v/v) APS and 0.1% (v/v) TEMED (Carl Roth, cat. no. 2367.2) immediately before use

### RNA loading buffer

99% (v/v) formamide (Sigma‐Aldrich, Merck, cat. no. 47671‐250ML‐F)
0.1% (w/v) bromophenol blue
0.1% (w/v) xylene cyanol
Store ≤1 year at room temperature

### SB medium

3.5% (w/v) tryptone
2% (w/v) yeast
0.5% (w/v) NaCl
1 N NaOH
Transfer into sterile 5‐L culture flasks
Autoclave
Prepare immediately before use
Add appropriate antibiotic before inoculation
  We use 100 µg/ml ampicillin.

### TA buffer (pH 8.2), 10×

500 mM Tris
1 M NaOAc
Adjust pH to 8.2 using NaOH (if too acidic), with final fine‐tuning using acetic acid
Store ≤6 months at 4°C

### TAE buffer (pH 8.0), 50×

2 M Tris
0.9 M acetic acid
10 mM EDTA
Adjust pH to 8.0 using NaOH (if too acidic), with final fine‐tuning using acetic acid
Store ≤1 year at room temperature

### TBE buffer (pH 8.0), 10×

0.9 M Tris
0.9 M boric acid
20 mM EDTA
Adjust pH to 8.0 using NaOH, as boric acid contributes to slightly acidic starting pH
Store ≤6 months at 4°C

### Tris/glutamate (pH 8.1), 1 M

1 M Tris in deionized H_2_O
Adjust pH to 8.1 using glutamic acid
Store ≤6 months at −20°C (in 1‐Pml aliquots)

## COMMENTARY

### Critical Parameters

#### Handling of data inputs used in virtual screening

The number and quality of pockets (receptors) identified and docked will form the basis of how long this set of protocols will take and how high quality your results are. If you have any *a priori* knowledge of where you would like your molecules to bind, use that when choosing which pocket(s) to include or exclude. If you have any knowledge of competing binding sites in other RNAs that may lower specificity, make sure to include those RNAs and their pockets as decoy structures in the selectivity cross‐docking step. Make sure the conformers you are selecting are sufficiently unique such that you are increasing your conformational search space. Conformers with a very low root mean square deviation (RMSD) relative to one another will create similar receptors that vastly increase docking time while only minimally boosting your search space.

#### RNA sample preparation

During the preparation of any *in vitro* RNA sample (Basic Protocol 4), working in an RNase‐free environment is crucial. Filter all buffers used for IVT and NMR measurements (Basic Protocols 4 and 5) freshly using sterile syringe filters.

### Troubleshooting

A troubleshooting guide is provided in Table 5.

**Table 5:** Troubleshooting Guide for Integrative *In Silico* and *In Vitro* Screening of Small Molecules Targeting RNA

Problem	Solution
No pockets being identified in the receptor generation process	If you run PocketFinder on default settings in ICM and it does not identify any pockets for many of the conformers, you can first try to decrease the tolerance value and re‐run PocketFinder. Usually, this problem occurs when the RNA is relatively small, with a simple helical structure. If this is the case, you can also select the entire RNA as the receptor instead of using the PocketFinder function.
Poor docking performance or unrealistic binding poses in virtual screening	Verify the quality and conformational diversity of the RNA structure. Use ensemble docking instead of a single structure and make sure the protonation states of the RNA and the ligands are correct before docking. If many of the scores are high (poor quality), you can also adjust the thoroughness parameter in the “Run_Docking” script. The default is set to 1, but you can increase it to allow for a longer search time (more Monte‐Carlo iterations) in the docking process. This will increase your docking time but allows for a higher chance of the ligand finding a favorable binding pose in the receptor.
Incorrect protonation states of ligands affecting docking or binding	Adjust the protonation state of the ligand according to the experimental buffer conditions (e.g., pH 6.2). Prior to docking and NMR experiments, use the appropriate mentioned software tools to assign protonation states.
Low RNA yield after IVT	Optimize the transcription conditions, such as the Mg^2+^ and NTP ratios and the template concentration. Consider adding DMSO to reduce template secondary structure and improve polymerase processivity.
RNA adopts incorrect or heterogeneous secondary structure in *in vitro* experiments (native PAGE and NMR)	Optimize the folding conditions (e.g., snap cooling vs. slow cooling). If necessary, add stabilizing ions, such as Mg^2+^, to encourage native folding, particularly for structured RNAs. Validate the folding process using native PAGE or NMR before screening.
RNA aggregation or dimerization at high concentrations	Reduce the RNA concentration or adjust the buffer conditions. Avoid concentrations that promote intermolecular interactions, such as concentrations >1 mM for certain constructs, like SL1.
Impurities or acrylamide contamination after PAGE purification	Perform additional RP‐HPLC purification and verify RNA purity by 1D‐^1^H NMR. Repeat purification if necessary.
Low solubility or precipitation of ligands after freeze‐thaw cycle(s)	Increase the DMSO concentration (e.g., up to 10‐15% if the RNA tolerates it) or prepare fresh stocks. Ensure gradual dilution into aqueous buffer to avoid precipitation. Some compounds might tolerate brief heating (1 min at 95°C) for dissolving. Check compound quality by recording a 1D‐^1^H spectrum.
Compound degradation due to improper storage or handling	Store compounds at –20°C and avoid repeated freeze‐thaw cycles. Prepare aliquots and minimize exposure to light, moisture, or air where applicable.
Air bubbles or poor shimming affecting spectral quality	Centrifuge NMR tubes prior to measurement and use robust water suppression methods (e.g., SOGGY). Optimize shimming on the water signal.
Weak or no signal in waterLOGSY experiments	Increase the number of scans or ligand concentration. Verify correct phase settings and ensure proper water suppression (e.g., SOGGY sequence).
Bias in computational analysis or descriptor interpretation	Use appropriate statistical tests (e.g., ANOVA) and ensure that there is a sufficient sample size for each group. Validate your findings using multiple descriptor types and clustering methods.

### Understanding Results

The workflow presented in this article integrates computational and experimental approaches for the identification and validation of RNA‐binding small molecules (Fig. 2). Figure 2 provides representative benchmark data that users can expect when successfully reproducing the described protocols.

#### Computational workflow and ligand selection (Figure 2A, panels 1 and 2)

Figure 2A, panel 1A, shows how RNA conformational ensembles are generated for VS. A key outcome at this stage is the presence of structurally diverse yet biologically relevant RNA conformations. Successful execution should produce multiple low‐energy conformers that differ in local pocket geometry while maintaining the overall fold. Insufficient conformational diversity may result in poor docking performance and lower hit rates.

Figure 2A, panel 1B, shows representative ligand structures and docking poses. Compounds that progress through the workflow should display chemically reasonable binding modes within defined RNA pockets without severe steric clashes. Unrealistic poses or highly strained conformations usually suggest problems with ligand preparation or target definition.

Figure 2A, panel 2, summarizes the tiered VS strategy. The distribution of docking scores (histogram) should clearly show an enrichment of top‐scoring compounds (Tier0 to Tier3). As filtering progresses, the number of compounds decreases while retaining structural diversity. Successful workflows result in a focused subset of candidates (Tier2 and Tier3) with favorable predicted binding properties. Insufficient scoring sensitivity or suboptimal target preparation may be indicated by a lack of score separation between tiers.

#### RNA preparation and validation (Figure 2B, panel 3)

Figure 2B, panel 3, shows the IVT and preparation of RNA samples. Successful RNA production yields a single dominant band in denaturing PAGE that corresponds to the expected transcript length. After combining PAGE and RP‐HPLC purification, the RNA should be free of major impurities, such as truncated products or residual acrylamide. In subsequent QC steps, native PAGE should indicate a homogeneous folding state (single band or defined conformer distribution), and 1D‐^1^H NMR spectra should show well‐dispersed imino and aromatic signals consistent with structured RNA.

#### NMR‐based screening and hit validation (Figure 2B, panel 4)

Figure 2B, panel 4, summarizes the results of representative NMR screening. Successful identification of RNA‐binding ligands depends on consistent observations across three orthogonal experiments:

i. 1D‐^1^H: Binding is indicated by CSPs and/or signal broadening relative to the free ligand;
ii. WaterLOGSY: Bound ligands produce positive signals, whereas unbound ligands remain negative;
iii. *T_2_*‐CPMG: Binding results in reduced signal intensity due to the RNA‐ligand complex's faster transverse relaxation.

Expected results include clear differences between ligand‐only‐ and RNA‐only‐containing samples. For strong binders, all three experiments typically show consistent effects. Weak binders may only show partial agreement across methods.

#### Binding‐site mapping and structural interpretation (Figure 2B, panel 4)

Binding‐site mapping via NMR uses CSPs of RNA resonances to provide residue‐specific information. Successful experiments yield localized CSP patterns that can be mapped onto the RNA structure to highlight potential ligand‐binding pockets.

#### Integration and prioritization of hits (Figure 2C, panel 5)

After experimental validation, the compounds are classified into binding categories, such as selective binders, dual binders, and non‐binders. Computational analysis of molecular descriptors and structural features should reveal trends that distinguish binders from non‐binders.

Typical observations include the following:

i. Enrichment of specific physicochemical properties (e.g., polarity, hydrogen bonding capacity) among binders;
ii. Recurring structural motifs identified via fingerprint analysis;
iii. Clustering chemically related compounds with similar binding behavior.

Failure to observe such trends may indicate an insufficient dataset size or high variability in binding modes.

### Time Considerations

Preparing RNA targets and ligand libraries usually takes up to 1 to 2 days for users familiar with ICM and Chemaxon software (Basic Protocol 1). Most of this time is spent on automated ligand protonation, energy minimization, conversion to ICM format, defining target pockets, and creating receptors based on those pockets. Docking a large diversity library of approximately 50,000 compounds against a single RNA conformer can take 10 to 15 days on a standard computer unit (Basic Protocol 2). To dock all the conformers in an ensemble and perform multiple replicates, parallelization over many CPUs is recommended to do so in a reasonable amount of time. Smaller libraries, such as those focused on RNA or FDA‐approved compounds, require proportionally less time (12 hr for a single conformer). Score aggregation and initial filtering take up to 8 hr. Redocking Tier1 compounds against the target and decoy RNA ensembles, followed by ensemble averaging, selectivity filtering, cross‐element docking, and visual inspection, typically takes 10 to 15 days, depending on the number of compounds and RNA elements analyzed and the number of CPUs available for the redocking steps. The procedure is largely automated, but manual inspection of poses may add time for QC. For the overall workflow duration of Basic Protocols 1 to 3, completing these protocols for a typical RNA target with a 50K compound library requires approximately 1 month, assuming you have at least 50 CPUs available for parallelization. Performing all three protocols on a single CPU would be prohibitively time consuming for a library of this size but could be reasonable for significantly smaller libraries.

Preparing ligand stocks and performing NMR‐based QC (Support Protocol) typically takes between 0.5 and 2 days, depending on the size of the library. For small libraries of fewer than 50 compounds, individual ^1^H spectra of each ligand are sufficient. For larger libraries of 100 to 200 compounds, pooling compounds with distinct NMR signatures into mixtures can reduce the acquisition time by ∼50%. Additional user time may be needed for compounds with poor solubility, including repeated measurements at higher DMSO concentrations. Preparing RNA targets for *in vitro* experiments usually takes 5 days (Basic Protocol 4). Most of this time is spent on plasmid transformation, bacterial culture, plasmid purification, linearization, IVT, and purification via denaturing PAGE and RP‐HPLC. QC of freshly synthesized RNA, including native PAGE and NMR checks, adds an additional 1 to 4 hr per construct. Using pre‐synthesized RNA from commercial vendors can substantially reduce preparation time, but verification of RNA integrity and folding is still required.

Setting up an NMR‐based screening of RNA‐ligand interactions (Basic Protocol 5) usually involves 1 to 2 days to prepare the samples, followed by 20 to 24 hr of automated NMR acquisition for each 96‐tube rack. The time required depends on the number of scans and the spectrometer parameters. Data processing and preliminary hit identification typically require an additional 1 day. Screening larger libraries may necessitate multiple measurement sessions; however, automated setups, such as the SampleJet™ system, help reduce hands‐on time. The characterization and prioritization of validated RNA‐binding hits (Basic Protocol 6) usually take 1 to 2 days. Classifying hits based on CSPs, *T_2_* reductions, and waterLOGSY factors takes 0.5 to 1 hr for a single compound set and 2 to 3 hr for 50 to 100 compounds. Computational preparation of ligand structures, calculation of molecular descriptors, fingerprint generation, and similarity clustering require 2 days depending on the size of the library and the available computational resources. Manual inspection of docking or clustering results may add time for QC.

The overall workflow duration for the Support Protocol and Basic Protocols 4 to 6 is approximately 8 to 10 days for a typical RNA target and a moderate ligand library of about 50 to 100 compounds. Using automated NMR acquisition and computational pipelines can substantially reduce hands‐on time and accelerate throughput.

### Author Contributions

**Sabrina Toews**: Conceptualization; data curation; formal analysis; investigation; methodology; software; visualization; writing—original draft. **Megan Ken**: Conceptualization; data curation; formal analysis; funding acquisition; investigation; methodology; project administration; resources; software; supervision; writing—review and editing. **Harald Schwalbe**: Conceptualization; funding acquisition; project administration; resources; supervision; writing—review and editing.

### Conflict of Interest

The authors declare that they have no competing interests.

## Data Availability

The data, tools, and material (or their source) that support the protocols are available from the corresponding authors upon reasonable request. The scripts related to the workflow presented here were deposited on GitHub and can be found via the following link: https://github.com/Ken‐Lab‐ScrippsResearch/Curr‐Protoc‐2026‐SARSCoV2.

## References

[cpz170431-bib-0001] Bereiter, R. , Flemmich, L. , Nykiel, K. , Heel, S. , Geley, S. , Hanisch, M. , Eichler, C. , Breuker, K. , Lusser, A. , & Micura, R. (2025). Engineering covalent small molecule–RNA complexes in living cells. Nature Chemical Biology, 21, 843–854. 10.1038/s41589-024-01801-3 39762536 PMC12122380

[cpz170431-bib-0002] Berg, H. , Wirtz Martin, M. A. , Niesteruk, A. , Richter, C. , Sreeramulu, S. , & Schwalbe, H. (2021). NMR‐based fragment screening in a minimum sample but maximum automation mode. Journal of Visualized Experiments, (172), e62262. 10.3791/62262 34152328

[cpz170431-bib-0003] Binas, O. , de Jesus, V. , Landgraf, T. , Völklein, A. E. , Martins, J. , Hymon, D. , Kaur Bains, J. , Berg, H. , Biedenbänder, T. , Fürtig, B. , Lakshmi Gande, S. , Niesteruk, A. , Oxenfarth, A. , Shahin Qureshi, N. , Schamber, T. , Schnieders, R. , Tröster, A. , Wacker, A. , Wirmer‐Bartoschek, J. , … Schwalbe, H. (2021). 19 F NMR‐based fragment screening for 14 different biologically active RNAs and 10 DNA and protein counter‐screens. ChemBioChem, 22, 423–433. 10.1002/cbic.202000476 32794266 PMC7436455

[cpz170431-bib-0004] Bonilla, S. L. , & Jang, K. (2024). Challenges, advances, and opportunities in RNA structural biology by cryo‐EM. Current Opinion in Structural Biology, 88, 102894. 10.1016/j.sbi.2024.102894 39121532

[cpz170431-bib-0005] Braunschweiler, L. , Bodenhausen, G. , & Ernst, R. R. (1983). Coherence transfer by isotropic mixing: Application to proton correlation spectroscopy. Molecular Physics, 48, 535–560. 10.1080/00268978300100381

[cpz170431-bib-0006] Cai, Z. , Ma, H. , Ye, F. , Lei, D. , Deng, Z. , Li, Y. , Gu, R. , & Wen, H. (2025). Discovery of RNA‐targeting small molecules: Challenges and future directions. MedComm, 6, e70342. 10.1002/mco2.70342 40859960 PMC12375692

[cpz170431-bib-0007] Ceylan, B. , Adam, J. , Toews, S. , Kaiser, F. , Dörr, J. , Scheppa, D. , Tants, J.‐N. , Smart, A. , Schoth, J. , Philipp, S. , Stirnal, E. , Ferner, J. , Richter, C. , Sreeramulu, S. , Caliskan, N. , Schlundt, A. , Weigand, J. E. , Göbel, M. , Wacker, A. , & Schwalbe, H. (2025). Optimization of structure‐guided development of chemical probes for the pseudoknot RNA of the frameshift element in SARS‐CoV‐2. Angewandte Chemie International Edition, 64, e202417961. 10.1002/anie.202417961 39887818

[cpz170431-bib-0008] Childs‐Disney, J. L. , Yang, X. , Gibaut, Q. M. R. , Tong, Y. , Batey, R. T. , & Disney, M. D. (2022). Targeting RNA structures with small molecules. Nature Reviews Drug Discovery, 21, 736–762. 10.1038/s41573-022-00521-4 35941229 PMC9360655

[cpz170431-bib-0009] Costales, M. G. , Childs‐Disney, J. L. , Haniff, H. S. , & Disney, M. D. (2020). How we think about targeting RNA with small molecules. Journal of Medicinal Chemistry, 63, 8880–8900. 10.1021/acs.jmedchem.9b01927 32212706 PMC7486258

[cpz170431-bib-0010] Dalvit, C. (1998). Efficient multiple‐solvent suppression for the study of the interactions of organic solvents with biomolecules. Journal of Biomolecular NMR, 11, 437–444. 10.1023/A:1008272928075

[cpz170431-bib-0011] Dalvit, C. , Fogliatto, G. P. , Stewart, A. , Veronesi, M. , & Stockman, B. (2001). WaterLOGSY as a method for primary NMR screening: Practical aspects and range of applicability. Journal of Biomolecular NMR, 21, 349–359. 10.1023/A:1013302231549 11824754

[cpz170431-bib-0012] Disney, M. D. (2019). Targeting RNA with small molecules to capture opportunities at the intersection of chemistry, biology, and medicine. Journal of the American Chemical Society, 141, 6776–6790. 10.1021/jacs.8b13419 30896935 PMC6541398

[cpz170431-bib-0013] Falese, J. P. , Donlic, A. , & Hargrove, A. E. (2021). Targeting RNA with small molecules: From fundamental principles towards the clinic. Chemical Society Reviews, 50, 2224–2243. 10.1039/D0CS01261K 33458725 PMC8018613

[cpz170431-bib-0014] Davis, D. G. , & Bax, A. (2004). Homonuclear Hartmann‐Hahn transfer with reduced relaxation losses by use of the MOCCA‐XY16 multiple pulse sequence. Journal of the American Chemical Society, 107, 2820–2821. 10.1021/ja00295a052 14675818

[cpz170431-bib-0015] Ganser, L. R. , Kelly, M. L. , Herschlag, D. , & Al‐Hashimi, H. M. (2019). The roles of structural dynamics in the cellular functions of RNAs. Nature Reviews Molecular Cell Biology, 20, 474. 10.1038/s41580-019-0136-0 31182864 PMC7656661

[cpz170431-bib-0016] Ganser, L. R. , Lee, J. , Rangadurai, A. , Merriman, D. K. , Kelly, M. L. , Kansal, A. D. , Sathyamoorthy, B. , & Al‐Hashimi, H. M. (2018). High‐performance virtual screening by targeting a high‐resolution RNA dynamic ensemble. Nature Structural & Molecular Biology, 25, 425–434. 10.1038/s41594-018-0062-4 PMC5942591 29728655

[cpz170431-bib-0017] Guillerez, J. , Lopez, P. J. , Proux, F. , Launay, H. , & Dreyfus, M. (2005). A mutation in T7 RNA polymerase that facilitates promoter clearance. Proceedings of the National Academy of Sciences, 102, 5958–5963. 10.1073/pnas.0407141102 PMC1087904 15831591

[cpz170431-bib-0018] Hajduk, P. J. , Olejniczak, E. T. , & Fesik, S. W. (1997). One‐dimensional relaxation‐ and diffusion‐edited NMR methods for screening compounds that bind to macromolecules. Journal of the American Chemical Society, 119, 12257–12261. 10.1021/ja9715962

[cpz170431-bib-0019] Hermann, T. (2016). Small molecules targeting viral RNA. WIREs RNA, 7, 726–743. https://doi/pdf/10.1002/wrna.1373 27307213 10.1002/wrna.1373 PMC7169885

[cpz170431-bib-0020] Ille, A. M. , Lamont, H. , & Mathews, M. B. (2022). The Central Dogma revisited: Insights from protein synthesis, CRISPR, and beyond. WIREs RNA, 13, e1718. 10.1002/wrna.1718 35199457

[cpz170431-bib-0021] Kang, C. , Nguyen, H. T. , Heppner, D. E. , Yu, B. , & Xu, W. (2026). Small‐molecule drug discovery targeting RNAs: Hope or hype? Journal of Medicinal Chemistry, 69, 1786–1789. 10.1021/acs.jmedchem.6c00070 41681135

[cpz170431-bib-0022] Kelly, J. A. , Woodside, M. T. , & Dinman, J. D. (2021). Programmed −1 ribosomal frameshifting in coronaviruses: A therapeutic target. Virology, 554, 75–82. 10.1126/science.aah3963 33387787 PMC7833279

[cpz170431-bib-0023] Koehn, J. T. , Felder, S. , & Weeks, K. M. (2023). Innovations in targeting RNA by fragment‐based ligand discovery. Current Opinion in Structural Biology, 79, 102550. 10.1016/j.sbi.2023.102550 36863268 PMC10023403

[cpz170431-bib-0024] Korb, O. , Olsson, T. S. G. , Bowden, S. J. , Hall, R. J. , Verdonk, M. L. , Liebeschuetz, J. W. , & Cole, J. C. (2012). Potential and limitations of ensemble docking. Journal of Chemical Information and Modeling, 52, 1262–1274. 10.1021/ci2005934 22482774

[cpz170431-bib-0025] Liu, G. , Du, W. , Sang, X. , Tong, Q. , Wang, Y. , Chen, G. , Yuan, Y. I. , Jiang, L. , Cheng, W. , Liu, D. , Tian, Y. , & Fu, X. (2022). RNA G‐quadruplex in TMPRSS2 reduces SARS‐CoV‐2 infection. Nature Communications, 13, 1444‐. 10.1038/s41467-022-29135-5 PMC8931161 35301316

[cpz170431-bib-0026] Meyer, S. M. , Williams, C. C. , Akahori, Y. , Tanaka, T. , Aikawa, H. , Tong, Y. , Childs‐Disney, J. L. , & Disney, M. D. (2020). Small molecule recognition of disease‐relevant RNA structures. Chemical Society Reviews, 49, 7167–7199. 10.1039/D0CS00560F 32975549 PMC7717589

[cpz170431-bib-0027] Nguyen, B. D. , Meng, X. , Donovan, K. J. , & Shaka, A. J. (2007). SOGGY: Solvent‐optimized double gradient spectroscopy for water suppression. A comparison with some existing techniques. Journal of Magnetic Resonance, 184, 263–274. 10.1016/j.jmr.2006.10.014 17126049

[cpz170431-bib-0028] Rangan, R. , Watkins, A. M. , Chacon, J. , Kretsch, R. , Kladwang, W. , Zheludev, I. N. , Townley, J. , Rynge, M. , Thain, G. , & Das, R. (2021). De novo 3D models of SARS‐CoV‐2 RNA elements from consensus experimental secondary structures. Nucleic Acids Research, 49, 3092–3108. 10.1093/nar/gkab119 33693814 PMC8034642

[cpz170431-bib-0029] Richter, C. , Hohmann, K. F. , Toews, S. , Mathieu, D. , Altincekic, N. , Bains, J. K. , Binas, O. , Ceylan, B. , Duchardt‐Ferner, E. , Ferner, J. , Fürtig, B. , Grün, J. T. , Hengesbach, M. , Hymon, D. , Jonker, H. R. A. , Knezic, B. , Korn, S. M. , Landgraf, T. , Löhr, F. , … Wacker, A. (2021). 1H, 13C and 15N assignment of stem‐loop SL1 from the 5’‐UTR of SARS‐CoV‐2. Biomolecular NMR Assignments, 15, 467–474. 10.1007/s12104-021-10047-2 34453696 PMC8401371

[cpz170431-bib-0030] Rückriegel, S. , Stamatakis, K. , Wachtveitl, J. , & Fürtig, B. (2025). Tiny but multi‐stable: Four distinct conformational states govern the ligand‐free state of the preQ1 riboswitch from a thermophilic bacterium. Nucleic Acids Research, 53, gkaf560. 10.1093/nar/gkaf560 40586310 PMC12207408

[cpz170431-bib-0031] Shah, R. , Yan, W. , Rigal, J. , Mullin, S. , Fan, L. , McGregor, L. , Krueger, A. , Renaud, N. , Byrnes, A. , & Thomas, J. R. (2025). Photoaffinity enabled transcriptome‐wide identification of splice modulating small molecule‐RNA binding events in native cells. RSC Chemical Biology, 6, 905–918. 10.1039/D4CB00266K 40226337 PMC11986670

[cpz170431-bib-0032] Shao, Y. , & Zhang, Q. C. (2020). Targeting RNA structures in diseases with small molecules. Essays in Biochemistry, 64, 955–966. 10.1042/EBC20200011 33078198 PMC7724634

[cpz170431-bib-0033] Shi, H. , Rangadurai, A. , Abou Assi, H. , Roy, R. , Case, D. A. , Herschlag, D. , Yesselman, J. D. , & Al‐Hashimi, H. M. (2020). Rapid and accurate determination of atomistic RNA dynamic ensemble models using NMR and structure prediction. Nature Communications, 11, 5531. 10.1038/s41467-020-19371-y PMC7608651 33139729

[cpz170431-bib-0034] Sreeramulu, S. , Richter, C. , Berg, H. , Wirtz Martin, M. A. , Ceylan, B. , Matzel, T. , Adam, J. , Altincekic, N. , Azzaoui, K. , Bains, J. K. , Blommers, M. J. J. , Ferner, J. , Fürtig, B. , Göbel, M. , Grün, J. T. , Hengesbach, M. , Hohmann, K. F. , Hymon, D. , Knezic, B. , … Schwalbe, H. (2021). Exploring the Druggability of Conserved RNA Regulatory Elements in the SARS‐CoV‐2 Genome. Angewandte Chemie International Edition, 60, 19191–19200. 10.1002/anie.202103693 34161644 PMC8426693

[cpz170431-bib-0035] Stelzer, A. C. , Frank, A. T. , Kratz, J. D. , Swanson, M. D. , Gonzalez‐Hernandez, M. J. , Lee, J. , Andricioaei, I. , Markovitz, D. M. , & Al‐Hashimi, H. M. (2011). Discovery of selective bioactive small molecules by targeting an RNA dynamic ensemble. Nature Chemical Biology, 7, 553–559. 10.1038/nchembio.596 21706033 PMC3319144

[cpz170431-bib-0036] Suresh, B. M. , Li, W. , Zhang, P. , Wang, K. W. , Yildirim, I. , Parker, C. G. , & Disney, M. D. (2020). A general fragment‐based approach to identify and optimize bioactive ligands targeting RNA. Proceedings of the National Academy of Sciences, 117, 33197–33203. 10.1073/pnas.2012217117 PMC7777249 33318191

[cpz170431-bib-0037] Taghavi, A. , Springer, N. A. , Zanon, P. R. A. , Li, Y. , Li, C. , Childs‐Disney, J. L. , & Disney, M. D. (2025). The evolution and application of RNA‐focused small molecule libraries. RSC Chemical Biology, 6, 510–527. 10.1039/D4CB00272E 39957993 PMC11824871

[cpz170431-bib-0038] Tants, J.‐N. , & Schlundt, A. (2024). The role of structure in regulatory RNA elements. Bioscience Reports, 44, BSR20240139. 10.1042/BSR20240139 39364891 PMC11499389

[cpz170431-bib-0039] Thompson, R. D. , Baisden, J. T. , & Zhang, Q. (2019). NMR characterization of RNA small molecule interactions. Methods, 167, 66–77. 10.1016/j.ymeth.2019.05.015 31128236 PMC6756962

[cpz170431-bib-0040] Toews, S. , Ceylan, B. , Wacker, A. , Ken, M. , & Schwalbe, H. (2025). Integrative in silico and in vitro screening of low molecular weight compounds targeting SARS‐CoV‐2 RNA elements. ChemBioChem, 26, e202500668. 10.1002/cbic.202500668 41317062 PMC12703450

[cpz170431-bib-0041] Toews, S. , Donà, F. , Keller, M. , Krauß, J. , Bracher, F. , López‐García, Ú. , Pabel, J. , Merk, D. , Blommers, M. J. J. , Ferner, J. , Wacker, A. , Richter, C. , & Schwalbe, H. (2025). Targeting the SARS‐CoV‐2 RNA translation initiation element SL1 by molecules of low molecular weight. Journal of the American Chemical Society, 147, 28783–28798. 10.1021/jacs.5c05264 40758647 PMC12356538

[cpz170431-bib-0042] Toews, S. , Wacker, A. , Faison, E. M. , Duchardt‐Ferner, E. , Richter, C. , Mathieu, D. , Bottaro, S. , Zhang, Q. , & Schwalbe, H. (2024). The 5′‐terminal stem–loop RNA element of SARS‐CoV‐2 features highly dynamic structural elements that are sensitive to differences in cellular pH. Nucleic Acids Research, 52, 7971–7986. 10.1093/nar/gkae477 38842942 PMC11260494

[cpz170431-bib-0043] Tong, Y. , Taghavi, A. , Su, X. , Yang, X. , Rouse, W. , Kovachka, S. , Childs‐Disney, J. L. , Sekioka, R. , Zavala, R. , Li, Y. , Moss, W. N. , & Disney, M. D. (2025). Live‐cell covalent profiling reveals principles of RNA‐small molecule recognition across the human transcriptome. bioRxiv, 2025.11.05.686775. 10.1101/2025.11.05.686775

[cpz170431-bib-0044] Veenbaas, S. D. , Koehn, J. T. , Irving, P. S. , Lama, N. N. , & Weeks, K. M. (2025). Ligand‐binding pockets in RNA and where to find them. Proceedings of the National Academy of Sciences, 122, e2422346122. 10.1073/pnas.2422346122 PMC12054788 40261926

[cpz170431-bib-0045] Wacker, A. , Weigand, J. E. , Akabayov, S. R. , Altincekic, N. , Bains, J. K. , Banijamali, E. , Binas, O. , Castillo‐Martinez, J. , Cetiner, E. , Ceylan, B. , Chiu, L.‐Y. , Davila‐Calderon, J. , Dhamotharan, K. , Duchardt‐Ferner, E. , Ferner, J. , Frydman, L. , Fürtig, B. , Gallego, J. , Grün, J. T. , … Zetzsche, H. (2020). Secondary structure determination of conserved SARS‐CoV‐2 RNA elements by NMR spectroscopy. Nucleic Acids Research, 48, 12415–12435. 10.1093/nar/gkaa1013 33167030 PMC7736788

[cpz170431-bib-0046] Zafferani, M. , & Hargrove, A. E. (2021). Small molecule targeting of biologically relevant RNA tertiary and quaternary structures. Cell Chemical Biology, 28, 594–609. 10.1016/j.chembiol.2021.03.003 33823146 PMC8141026

[cpz170431-bib-0047] Zhou, Y. , Jiang, Y. , & Chen, S. J. (2022). RNA‐ligand molecular docking: Advances and challenges. WIREs Computational Molecular Science, 12, e1571. 10.1002/wcms.1571 37293430 PMC10250017

